# Theranostic porphyrin nanoparticles identify atherosclerosis via multimodal imaging and elicit atheroprotective effects

**DOI:** 10.1016/j.mtbio.2025.102202

**Published:** 2025-08-21

**Authors:** Victoria A. Nankivell, Lauren Sandeman, Liam Stretton, Achini K. Vidanapathirana, Maneesha A. Rajora, Juan Chen, William Tieu, Hanyi Weng, Maaike Kockx, Leonard Kritharides, Peter J. Psaltis, Joanne T.M. Tan, Yung-Chih Chen, Karlheinz Peter, Gang Zheng, Christina A. Bursill

**Affiliations:** a Vascular Research Centre, Lifelong Health Theme, South Australian Health and Medical Research Institute, Adelaide, South Australia, 5000, Australia; b Australian Research Council (ARC) Centre of Excellence for Nanoscale BioPhotonics (CNBP), Australia; c Faculty of Health and Medical Science, Adelaide Medical School, University of Adelaide, Adelaide, South Australia, 5000, Australia; d Princess Margaret Cancer Centre, University Health Network, Toronto, Ontario, M5G 1L7, Canada; e Department of Medical Biophysics, University of Toronto, Ontario, M5G 1L7, Canada; f ANZAC Research Institute, Concord Repatriation General Hospital, Sydney Local Health District and University of Sydney, Australia; g Department of Cardiology, Concord Repatriation General Hospital, Sydney Local Health District and University of Sydney, Australia; h Atherothrombosis and Vascular Biology, Baker Heart and Diabetes Institute, Melbourne, Victoria, Australia

**Keywords:** Lipid-based nanoparticles, Imaging atherosclerosis, Multimodal imaging, Anti-inflammatory, Porphyrin-lipid

## Abstract

**Background:**

Porphyrin-lipid nanoparticles (Por-NPs) have unrealized potential for atherosclerosis. Por-NPs incorporate porphyrin-lipid which permits fluorescence imaging and chelates Copper-64 (^64^Cu) for positron emission tomography (PET) imaging. Their outer shell contains a short peptide ‘R4F’ that enables macrophage targeting and therapeutic effects. Accordingly, this study investigates the simultaneous diagnostic and therapeutic properties of Por-NPs in atherosclerosis.

**Results:**

*In vitro*, Por-NPs were found to be internalized by immortalised bone marrow-derived macrophages (iBMDMs), visualized via fluorescence microscopy and flow cytometry. Por-NPs also increased cholesterol efflux from [^3^H]-cholesterol-loaded iBMDMs, (49 %, *P* < 0.05). Incubation of iBMDMs with Por-NPs reduced mRNA levels of inflammatory mediators *Il1b* (88 %), *Il18* (54 %) *Ccl5* (75 %) and *Ccl17* (92 %), and protein secretion of IL-1β (69 %), CCL5 (82 %) and CCL17 (94 %), *P* < 0.05. Por-NPs suppressed inflammasome components *Nlrp3* (69 %) and *Asc* (36 %), *P* < 0.05. Studies using siRNA deletion of SR-B1 and methyl-β-cyclodextrin, revealed the anti-inflammatory properties of Por-NPs were independent of SR-B1 and cholesterol efflux. However, Por-NPs suppressed activation of inflammatory transcription factor NF-κB (53 %, *P* < 0.05). *In vivo*, in *Apoe*^−/−^ mice, serial non-invasive PET imaging showed ^64^Cu-labelled Por-NPs localised in hearts and detected increases in plaque size longitudinally with high-cholesterol diet. Por-NP fluorescence was visualized in aortic sinus plaques, co-localised with CD68^+^ macrophages, and by fluorescence IVIS imaging in aortic arch plaque. In two murine models, Por-NP-treated mice had smaller early-stage (22 %) and unstable plaques (52 %). Por-NP-treated mice had fewer circulating (32 %) and aortic (81 %) monocytes, and lower mRNA levels of aortic arch *Rela* (26 %) and *Nfkb1* (27 %), *P* < 0.05.

**Conclusions:**

Por-NPs detect plaques using multiple imaging modalities and exhibit atheroprotective effects, presenting as novel nanoscale theranostics for atherosclerosis.

## Highlights

• Atherosclerosis is the deposition of fatty plaques in arteries and the main cause of heart attacks.
• Strategies to improve detection and reduce atherosclerotic plaques are needed.
• Multifunctional porphyrin-lipid nanoparticles (Por-NPs) have diagnostic and therapeutic properties.
• Por-NPs are internalized by macrophages and track to atherosclerotic plaques, visualized using multiple imaging modalities.
• Por-NPs exert atheroprotective effects *in vitro* in macrophages. They suppress inflammation and promote cholesterol efflux.
• Por-NPs exhibit therapeutic effects in two murine models of atherosclerosis and significantly reduce plaque size.

## Graphical abstract

**Figure:**
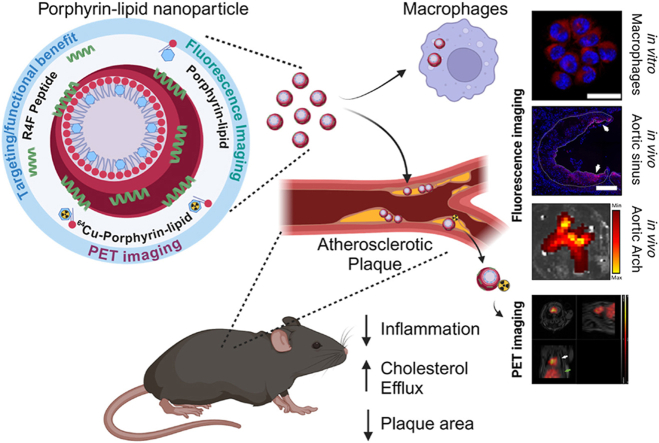

## 1 Introduction

Atherosclerosis, the deposition of fatty plaque in the arteries, is driven by inflammation and lipid accumulation [1]. Despite substantial improvements in evidence-based preventative approaches, there remains an unacceptably high number of people who suffer cardiovascular complications as a result of atherosclerotic occlusions [2]. This illustrates the need for novel therapies and diagnostics that improve the management of atherosclerotic cardiovascular disease (CVD).

Current therapeutic and diagnostic strategies for atherosclerotic disease focus on lipid-lowering therapy and angiographic techniques. These are effective but can be insufficient for early disease detection, which is an important part of preventing major adverse cardiac events such as myocardial infarction and stroke. There is an increasing body of research demonstrating the potential of developing multifunctional nanoparticles for applications in atherosclerotic CVD, in particular those that target plaque macrophages [3]. Nanoparticles are an exciting technology that can be designed to target atherosclerotic plaques and provide diagnostic or therapeutic effects [[3], [4], [5], [6]]. Their greatest potential lies in strategies that combine therapeutic and diagnostic utility into a single nanoparticle. A nanoparticle with the ability to enable multimodal plaque imaging and elicit pleiotropic atheroprotective actions including cholesterol efflux and the inhibition of inflammation, holds promise as an effective theranostic for atherosclerosis [3,7].

Porphyrin-lipid nanoparticles (Por-NPs) are an organic lipid-based nanoparticle with multimodal imaging and therapeutic capabilities and have several features that set them apart. The structure of Por-NPs consists of porphyrin-lipid conjugates within the outer lipid shell simultaneously enabling excellent near-infrared fluorescence and serial non-invasive PET imaging capabilities through chelation of radionuclides such as Copper-64 (^64^Cu) [[8], [9], [10]]. Por-NPs also incorporate a short apoA-I mimetic peptide ‘R4F’ (Ac-FAEKFKEAVKDYFAKFWD) that constrains the lipid shell to ∼20 nm in diameter via an α-helix network structure that facilitates cellular uptake [11]. Our previous research on cancer theranostics has demonstrated the biosafety and biocompatibility of these porphyrin nanoparticles [[11], [12], [13], [14]]. Importantly, R4F allows for cell targeting and therapeutic capabilities via pathways that have benefit for atherosclerosis applications. The R4F structure interacts with the scavenger receptor class B type I (SR-BI), which is highly expressed on atherosclerotic plaque macrophages and mediates cell signaling events associated with cholesterol efflux/influx and inflammation [[15], [16], [17]]. Whilst previously explored for targeted cancer therapy and diagnostics [11,13], Por-NPs have intriguing potential for atherosclerosis.

Accordingly, this study tested the ability of Por-NPs to detect and reduce atherosclerosis. *In vitro*, we found Por-NPs are internalized by macrophages, promote cholesterol efflux and inhibit inflammation. *In vivo*, infusions of Por-NPs in atherosclerosis-prone apolipoprotein (*Apo)e*^−/−^ mice fed a high-cholesterol diet were found to track to atherosclerotic plaques, visualized using both fluorescence and serial PET imaging. Furthermore, mice injected with Por-NPs developed smaller atherosclerotic plaques, demonstrating atheroprotective properties *in vivo*. These findings have implications for the simultaneous identification and prevention of atherosclerosis using multifunctional Por-NP nanoscale theranostics.

## 2 Methods

### 2.1 Porphyrin-lipid nanoparticles

The discoidal and cholesterol oleate (CO)-loaded porphyrin-lipid nanoparticles (Por-NPs) were synthesized and characterized as per Supplemental Methods for nanoparticle synthesis. Table S1 summarizes nanoparticle compositions.

### 2.2 ^64^Cu-labelling of Por-NPs

^64^Cu-Cl_2_ production and nanoparticle labelling were conducted by the Molecular Imaging and Therapy Research Unit (MITRU, SAHMRI, Adelaide). Por-NPs were labelled as previously described [9,11]. Radiochemical purity was verified by thin layer chromatography and activity measured prior to intravenous injection.

### 2.3 Cell culture

Immortalised murine bone marrow derived macrophages (iBMDMs) were kindly provided by Dr Ashley Mansell (Hudson Institute of Medical Research, Victoria, Australia). iBMDMs were originally isolated from C57BL/6 mouse bone marrow, then differentiated into macrophages prior to being immortalised using the Cre-J2 retroviral infection method. iBMDMs were maintained in low glucose DMEM (D5523, Sigma) supplemented with 10 % FBS (Sigma) in a 37^o^C, 5 % CO_2_ incubator. Cells were seeded 24 h prior to treatments. Tet (tetracycline)-inducible human SR-BI Chinese Hamster Ovary (hSR-BI CHO) cells were maintained in Ham's F12 Nutrient Mix (11765054, Thermo-Fisher) supplemented with 10 % FBS, Penicillin (100 IU/mL), streptomycin (100 μg/mL), Blasticidin S (10 μg/mL) and Zeocin (400 μg/ml) in a 37^o^C, 5 % CO_2_ incubator. The stable monoclonal Tet-inducible hSR-BI CHO cells were generated by subcloning of hSR-B1 (NM-005505) in pCMV6-XL5 (OriGene Technologies) into pcDNA™4/TO (Life technologies) followed by transfection into T-RExTM- CHO cells (Life technologies). To induce hSR-BI expression, CHO cells were incubated with 1 μg/mL tetracycline for 18h and SR-BI overexpression confirmed by Western blot.

### 2.4 Por-NP fluorescence detection *in vitro*

iBMDMs were incubated with PBS, discoidal or (CO)-loaded Por-NPs (10 μg/mL, R4F concentration) for 24 h iBMDMs on microscope slides were mounted with VECTASHIELD® antifade medium with DAPI (Vector Laboratories) and porphyrin-lipid fluorescence detected by confocal microscopy (Leica, excitation λ: 653 nm; emission λ: 670 nm beyond). Por-NP uptake was analyzed by flow cytometry on a BD LSRFortessa™ X-20 Analyzer (Becton Dickinson) with the red laser (excitation λ: 640 nm; emission λ: 670/30 nm) to detect porphyrin-lipid fluorescence. Uptake of Por-NPs in iBMDM and hSR-BI CHO cells was quantified by measuring intracellular porphyrin fluorescence. Treated cells (PBS or Por-NPs, 3h) were washed 3 times in PBS, pelleted and resuspended in cell lysis buffer. Lysates were measured on Glomax plate reader (Promega) with excitation and emission wavelength 405 nm and 660–720 nm respectively. Cells grown on coverslips were imaged with fluorescence microscope (Nikon) with Cy5 and DAPI filters.

### 2.5 Cholesterol efflux

iBMDMs were incubated with 0.074 MBq/mL of ^3^H-cholesterol ([1,2–3H(N)]-labelled cholesterol, Perkin-Elmer) for 24 h at 37 °C, then with PBS, the cholesterol acceptor reconstituted high-density lipoprotein (rHDL, 25 μg/mL, apoA-I concentration), and discoidal or CO-loaded Por-NPs (10 or 25 μg/mL, R4F concentration) for 4 h. In some experiments the non-specific cholesterol acceptor methyl-β-cyclodextrin (MβCD, 4 % v/v) was included. Radioactivity of ^3^H-cholesterol was measured as counts per minute (CPM) in the supernatant and cell lysates with a liquid scintillation analyzer (Tricarb-2810TR, Perkin Elmer) then calculated as % cholesterol efflux: Supernatant CPM ÷ (Supernatant CPM + Cell lysate CPM).

### *2.6* *In vitro* inflammation

iBMDMs were incubated with PBS, discoidal or CO-loaded Por-NPs (10 μg/mL, R4F concentration) for 18 h and then stimulated with lipopolysaccharide (LPS, 10 ng/mL, L4391 Sigma) or murine interferon (IFN)-γ (10 ng/mL, 315-05 Peprotech) for 16 h.

### 2.7 Real time-qPCR

RNA was extracted from treated iBMDMs and murine aortic arches with TRI-reagent (Sigma-Aldrich) then reverse transcribed to cDNA using iScript Reverse Transcriptase Supermix (Biorad). Primers for inflammatory and housekeeper genes (Table S2) measured gene expression changes by qPCR, calculated using the ^ΔΔ^*Ct* method referenced to housekeeper genes *Rplp0* or *Beta-2 microglobulin* (*B2m).*

### 2.8 ELISAs

Secreted proteins were measured in cell culture supernatants from treated iBMDMs and in mouse plasma using ELISAs for IL-1β, CCL5 and CCL17 (Quantikine®, R&D systems).

### 2.9 siRNA knockdown of SR-BI

iBMDMs were incubated with transfection reagent alone (Lipofectamine® RNAiMAX, 13778150, Thermofisher), scrambled control siRNA (sc-37007, Santa Cruz) or SR-BI siRNA (sc-44753, Santa Cruz) in optiMEM at 100 nM of siRNA for 6 h at 37^o^C. Media was replaced and cells incubated for a total of 48 h iBMDMs were incubated with PBS or discoidal Por-NPs (10 μg/mL, R4F concentration, 24 h) then stimulated with LPS for 16 h for inflammatory gene assessments.

### 2.10 Western blotting

Western blotting measured nuclear NF-κB p65 and SR-BI. For nuclear NF-κB p65, nuclear protein was isolated using the NE-PER nuclear kit (Thermo Scientific), then proteins separated on 4–12 % Bis-Tris Bolt gels using electrophoresis before transfer to nitrocellulose membranes (Thermo Fisher Scientific) by iBlot (Invitrogen). Membranes were incubated with rabbit anti-NF-κB p65 antibody [E379] (1:1000, ab32536, Abcam) followed by goat anti-rabbit HRP-conjugated antibody (1:2000). An anti-rabbit antibody against TATA-binding protein (1:1000, ab63766, Abcam) was the nuclear protein loading control. To validate siRNA SR-BI knockdown and overexpression in hSR-BI CHO cells, membranes were incubated with anti-SR-BI antibody [EP1556Y] (1:1000, ab52629, Abcam) followed by goat anti-rabbit HRP-conjugated antibody (1:2000). An anti-alpha tubulin antibody [DM1A] (1:1000, ab7291, Abcam) followed by goat anti-mouse HRP-conjugated antibody (1:1000) were used for protein loading control.

### 2.11 Animal care

All experiments and procedures were approved by the South Australian Health and Medical Research Institute (SAHMRI) Animal Ethics Committee (AEC application SAM422.19), conformed to the National Health and Medical Research Council (NHMRC) Australian code for the care and use of animals for scientific purposes and National Institutes of Health (NIH) Guide for the Care and Use of Laboratory Animals. Male C57Bl6/J apolipoprotein (*Apo*)*e*^−/−^ mice bred at SAHMRI were fed high cholesterol diet (HCD) containing 21 % fat and 0.15 % cholesterol (SF-00219, Semi-Pure Rodent Diet, Specialty Feeds) or standard rodent chow.

For early-stage stable atherosclerosis, 6-week-old mice were fed HCD for a total of 6 weeks. They were infused with Por-NPs (40 mg/kg, per R4F, ∼250 μl) or PBS at the start of the HCD-feeding period, 3-times per week via intraperitoneal injection until animals were humanely killed. For mid-late-stage stable atherosclerosis, 6-week-old mice were fed HCD for a total of 12 weeks. For the unstable plaque model, 12-week-old mice were fed HCD for a total of 13 weeks. The mice received Por-NPs (40 mg/kg, per R4F, ∼250 μl) or PBS via intraperitoneal injection immediately following the surgical intervention and then on alternate days until the end of the study 7-weeks later.

For both studies, mice were circulatory perfusion flushed with PBS immediately following humane killing via cardiac puncture. Whole blood and tissues were collected within 24 h following the final infusions of treatments. Mice were randomized into PBS or discoidal Por-NPs treatment groups. Blinding was used via random number allocation for histological and flow cytometric analyses.

### 2.12 Tandem stenosis surgery

After 6 weeks of HCD, mice received tandem stenosis carotid artery ligation surgery as described previously [18]. Briefly, animals were anaesthetised a single time by inhalation of 5 % isoflurane and maintained on 2–3 % isoflurane for the duration of the procedure. Tandem stenoses were made in the right common carotid artery with an outer diameter of 150 μm, separated by 3 mm, using 6-0 braided polyester sutures. Average duration of the procedure was 45 min. The placement of the sutures causes changes in blood flow dynamics that trigger the development of a plaque with unstable features including intraplaque haemorrhage [18].

After surgery, mice received PBS or discoidal Por-NPs (40 mg/kg, R4F concentration) via intraperitoneal injection on alternate days for seven weeks until animals were humanely killed.

### 2.13 ^64^Cu-Por-NP γ-counting for biodistribution assessment

*Apoe*^*−/−*^ mice were fed a HCD or chow for 32 weeks. Mice were administered ^64^Cu-labelled 30 mol % Por-NPs (∼18.5 MBq) and sacrificed 48 h post-injection. γ-Counts were measured from tissues and calculated as per: Cu64γcounts(sample)Cu64γcounts(totalinjecteddose)X100%Tissueweight(g)

### 2.14 Positron emission tomography (PET)/magnetic resonance imaging (MRI)

Infusions of ^64^Cu-Por-NPs (18.5–20 MBq) were administered intravenously into the mid-stage stable atherosclerosis cohort of *Apoe*^*−/−*^ mice at week 7 and 11 of HCD feeding. In the tandem stenosis model cohort, animals were imaged after 12 weeks of HCD feeding. Mice were imaged 6 h post-injection with a 5 min scan using the Albira PET (Bruker, MA, USA). PET images were quantified by PMOD v3.509 software (PMOD technologies LLC, Zurich, Switzerland). Volume of interest (VOI) regions were defined using VOI tool. For MRI, mice were imaged with an ICON 1T MRI (Bruker) for anatomical reference to complement PET imaging. For all live-animal imaging procedures, mice were maintained under isoflurane anaesthesia and scans did not exceed 30 min.

### 2.15 IVIS fluorescence imaging *ex vivo*

Tissues were harvested 24 h after a final intraperitoneal injection of PBS or Por-NP at 5 mg/kg (per porphyrin-lipid) for imaging on the In Vivo Imaging System (IVIS) Spectrum (PerkinElmer, MA, USA) at excitation and emission wavelengths 670 nm and 720 nm, respectively. Epi-fluorescence images were acquired with auto-exposure, F/stop = 2 and smallest binning settings with a field of view of 6.6–23 cm. Imaging of Por-NP Chow and HCD aortic arches and descending aortas were captured side-by-side. Images were analyzed for fluorescence in regions of interest (ROI) with Aura imaging software (Spectral Instruments Imaging, AZ, USA).

### 2.16 Histology

Fresh frozen sections of aortic sinus and tandem stenosis Segment I were stained with H&E (plaque area) and Oil red O (lipid). Plaque smooth muscle cells (α-SMA^+^) and macrophages (CD68^+^) were detected by immunofluorescence, and erythrocytes (TER-119^+^) by immunohistochemistry. Sections were imaged under brightfield or fluorescence microscopy and porphyrin-lipid detected with Cy5 filter. Plaque area was calculated from H&E-stained sections. For histological and immunochemical analyses, positive staining was calculated as a percentage of plaque area. See Supplemental Methods for further detail.

### 2.17 Flow cytometry of aorta and blood

Aortas were digested in Liberase TM (Roche) and passed through a cell strainer (40 μm) then incubated with fluorochrome-conjugated antibodies (Table S3) to detect: macrophages (CD11b^+^F4/80^+^) and M1 (CD86^+^CD206^-^)/M2 (CD86^−^CD206^+^) phenotypes, monocytes (CD11b^+^F4/80^-^), endothelial cells (CD11b^−^ F4/80^-^ CD31^+^) and smooth muscle cells (CD11b^−^F4/80^−^α-SMA^+^). For blood, taken by tail vein nick in the final week of treatment in early-stage group, we assessed: neutrophils (CD45^+^ CD11b^+^Gr-1^+^Ly6G^+^), monocytes (CD45^+^CD11b^+^CCR2^+^) and activated monocytes (CD45^+^CD11b^+^CCR2^+^Ly6C^hi^). Porphyrin-lipid^+^ cells were detected on the AF647 channel (excitation 640 nm, emission 670/30 nm). Viability dye (AF700) was used for all samples.

Samples were run on a BD LSRFortessa™ X-20 Analyzer and data analyzed with FlowJo™ v10.8.1 software. Fluorescence minus one (FMO) samples were used to set gates with porphyrin-lipid fluorescence gated using PBS controls.

### 2.18 Statistical analysis

All data expressed as Mean ± SD. Data with groups of more than two were analyzed by one-way ANOVA with post-hoc Tukey's multiple comparisons. Data with groups of two were analyzed by a two-tailed unpaired *t*-test, with exception of longitudinal PET activity quantification analyzed using a two-tailed paired *t*-test. Significance was defined as *P* < 0.05.

## 3 Results

### 3.1 Por-NPs and their internalization by macrophages

**Fig. 1:**
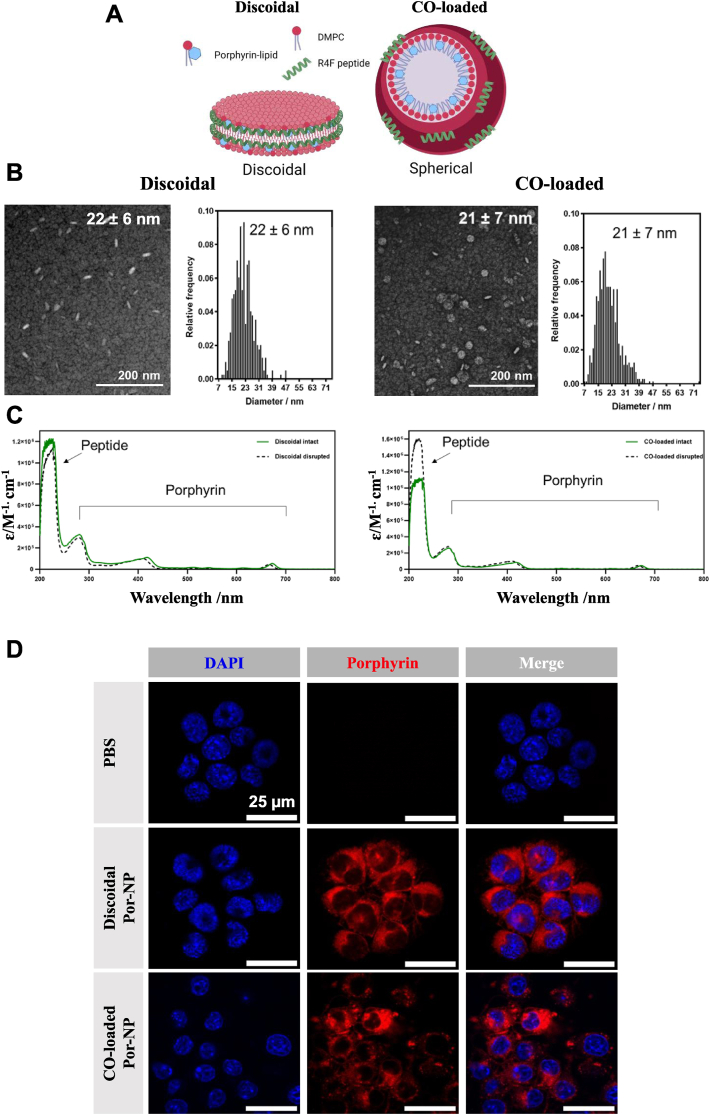
Por-NPs and their internalization by macrophages *in vitr*o. **(A)** Components and structures of discoidal and CO-loaded Por-NPs. **(B)** Transmission electron microscopy (TEM) of discoidal and CO-loaded Por-NPs with size distribution quantified from TEM. **(C)** Absorbance measured at 200–800 nm of either intact (green) or disrupted (with methanol, dashed) for discoidal and CO-loaded nanoparticles. **(D)** Confocal microscopy images of iBMDMs treated with PBS, discoidal Por-NPs or CO-loaded Por-NPs with porphyrin-lipid detected at 653 nm excitation. Scale bar: 25 μm. DMPC, 1,2-Dimyristoyl-sn-glycero-3-phosphocholine. (For interpretation of the references to colour in this figure legend, the reader is referred to the Web version of this article.)

We synthesized and tested discoidal and spherical-like cholesterol oleate (CO)-loaded Por-NPs. Particle size and shape were verified by transmission electron microscopy (TEM, Fig. 1A–B) that shows the spherical solid core morphology of the CO-loaded Por-NPs, which is distinct from a liposomal-based bilayer structure. Optical characterization of each Por-NP absorbance spectra yielded a slight red shift of 7 nm (from 667 nm to 674 nm) in intact compared to methanol-disrupted particles (Fig. 1C), consistent with previous observations [19]. This shift in absorbance spectra is associated with the assembly of porphyrin-lipid into intact nanoparticles. Additionally, the absorption spectra and mass spectrometry characterisation are in Supplemental Fig. S1. Incubation of iBMDMs with discoidal and CO-loaded Por-NPs *in vitro* led to robust uptake for both. Por-NPs were visualized within iBMDMs via porphyrin-lipid fluorescence detectable in the cytoplasm of macrophages and absent in the PBS-treated vehicle control (Fig. 1D). Por-NP uptake was also measurable by flow cytometry (670/30 nm channel, Supplemental Fig. S2A–B). iBMDMs incubated with discoidal Por-NPs exhibited a substantial increase in median fluorescence intensity of the porphyrin-lipid, compared to PBS controls (Supplemental Fig. S2C, *P* < 0.0001). Additionally, we also confirmed that overexpression of hSR-BI increased the uptake of Por-NPs in tetracycline-inducible hSR-BI CHO cells (Supplemental Fig. S3). Treatment with tetracycline induced SR-BI protein expression not seen in the untreated cells (Fig. S3A). This also led to increased uptake detected by porphyrin-lipid fluorescence in hSR-BI CHO cells (Fig. S3B–C).

### 3.2 Por-NPs promote cholesterol efflux and inhibit inflammation in macrophages

**Fig. 2:**
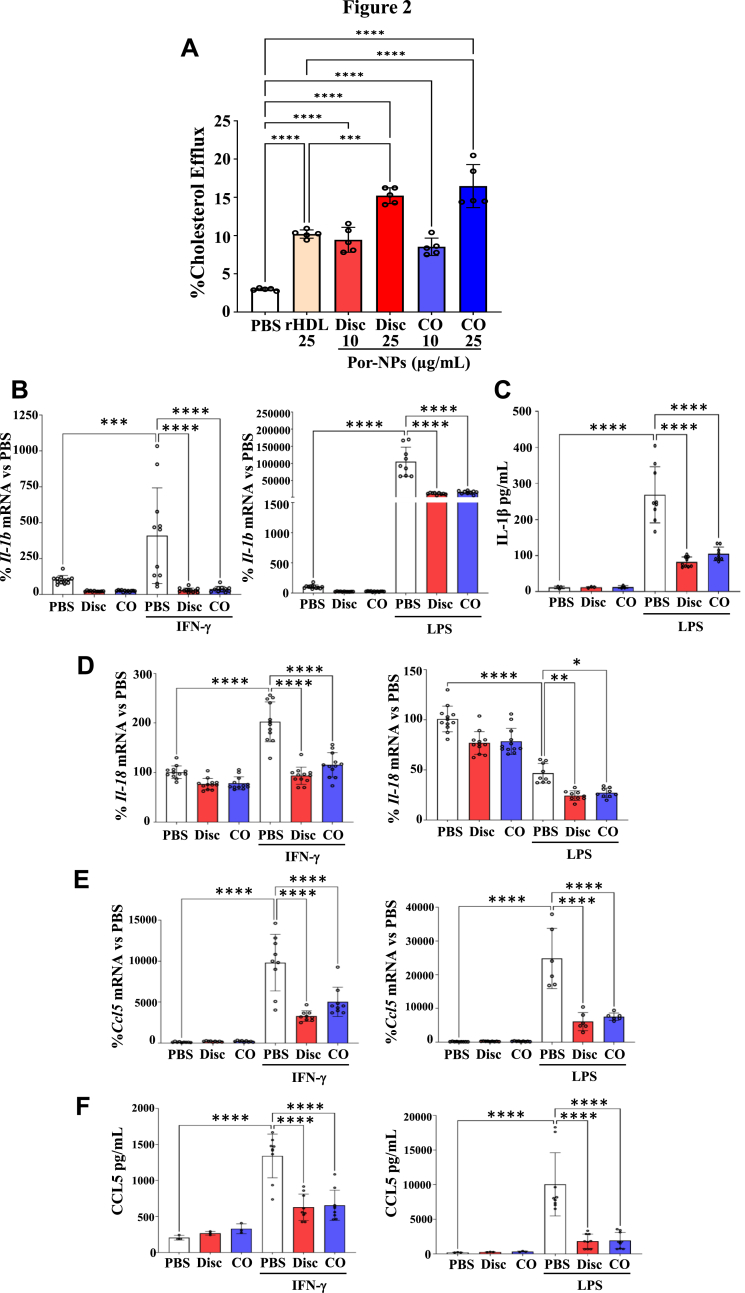
Por-NPs promote cholesterol efflux and inhibit inflammatory mediators in macrophages *in vitro*. (A**)** Cholesterol efflux measured in [^3^H]-cholesterol-loaded iBMDMs following treatment with either PBS vehicle control, rHDL, discoidal (Disc) or CO-loaded (CO) Por-NPs (n = 5 biological replicates). **(B)** RT-qPCR measurement of *Il1b* mRNA levels following incubation with discoidal (Disc) or CO-loaded (CO) Por-NPs and stimulation with IFN-γ (10 ng/mL) or LPS (10 ng/mL) and **(C)** ELISA measurement of secreted IL-1β protein in culture media. Following stimulation with IFN-γ and LPS, RT-qPCR assessment of **(D)***Il18* and **(E)***Ccl5* mRNA (n = 9–12 biological replicates) and, **(F)** by ELISA, secreted CCL5 protein (n = 3–9 biological replicates). Data expressed as Mean ± SD. ∗*P* < 0.05, ∗∗*P* < 0.01, ∗∗∗*P* < 0.001, ∗∗∗∗*P* < 0.0001 by one-way ANOVA with post-hoc Tukey's multiple comparisons.

SR-BI has been shown previously to facilitate cholesterol efflux [15,17,20]. Given that the R4F component of Por-NPs has been shown to target SR-BI [11,15,20], we tested the cholesterol efflux capacity of Por-NPs in iBMDMs. Compared to PBS control, incubation with discoidal and CO-loaded Por-NPs at 10 μg/mL increased cholesterol efflux (Fig. 2A, Disc: +219 %; CO: +189 %, *P* < 0.0001). Incubation with 25 μg/mL of Por-NPs led to further stepwise increases in cholesterol efflux compared to PBS control (Fig. 2A, Disc: +415 %; CO: +457 %, *P* < 0.0001). Interestingly, 25 μg/mL of Por-NPs elicited more cholesterol efflux than 25 μg/mL of rHDL (Fig. 2A, Disc: +49 %, *P* < 0.001; CO: +61 %, *P* < 0.0001).

We next determined the anti-inflammatory effects of Por-NPs in iBMDMs. As expected, IFN-γ and LPS significantly increased *Il1b* (*P* < 0.0001, Fig. 2B). This elevation was attenuated following incubation with discoidal Por-NPs (IFN-γ: 92 %, LPS: 88 %, *P* < 0.0001) and CO-loaded Por-NPs (IFN-γ: 91 %, LPS: 85 %, *P* < 0.0001). Consistent with this, LPS-stimulated secreted IL-1β protein levels were significantly lower in the culture media of iBMDMs incubated with Por-NPs (Fig. 2C, Disc: 69 %; CO: 61 %, *P* < 0.0001).

iBMDMs preincubated with discoidal and CO-loaded Por-NPs displayed significantly lower *Il1*8 mRNA levels than IFN-γ stimulated PBS controls (Fig. 2D, Disc: 54 %; CO: 43 %, *P* < 0.0001). Although incubation of iBMDMs with LPS caused an unexpected decrease in *Il18* (Figs. 2D, −54 %, *P* < 0.001), treatment with both discoidal and CO-loaded Por-NPs caused an additional decrease in *Il1*8 mRNA expression (Fig. 2D, Disc: 48 %, *P* < 0.01; CO-loaded: 42 %, *P* < 0.05).

Por-NPs reduced mRNA levels of chemokine *Ccl5* in iBMDMs for both discoidal (IFN-γ: 66 %; LPS: 75 %, *P* < 0.0001) and CO-loaded (IFN-γ: 49 %; LPS: 70 %, *P* < 0.0001) Por-NPs, compared to stimulated PBS controls (Fig. 2E). Por-NPs also decreased secreted CCL5 protein in culture media following stimulation with IFN-γ (Disc: 53 %; CO: 51 %, *P* < 0.0001) and LPS (Disc: 82 %; CO: 81 %, *P* < 0.0001) (Fig. 2F).

**Fig. 3:**
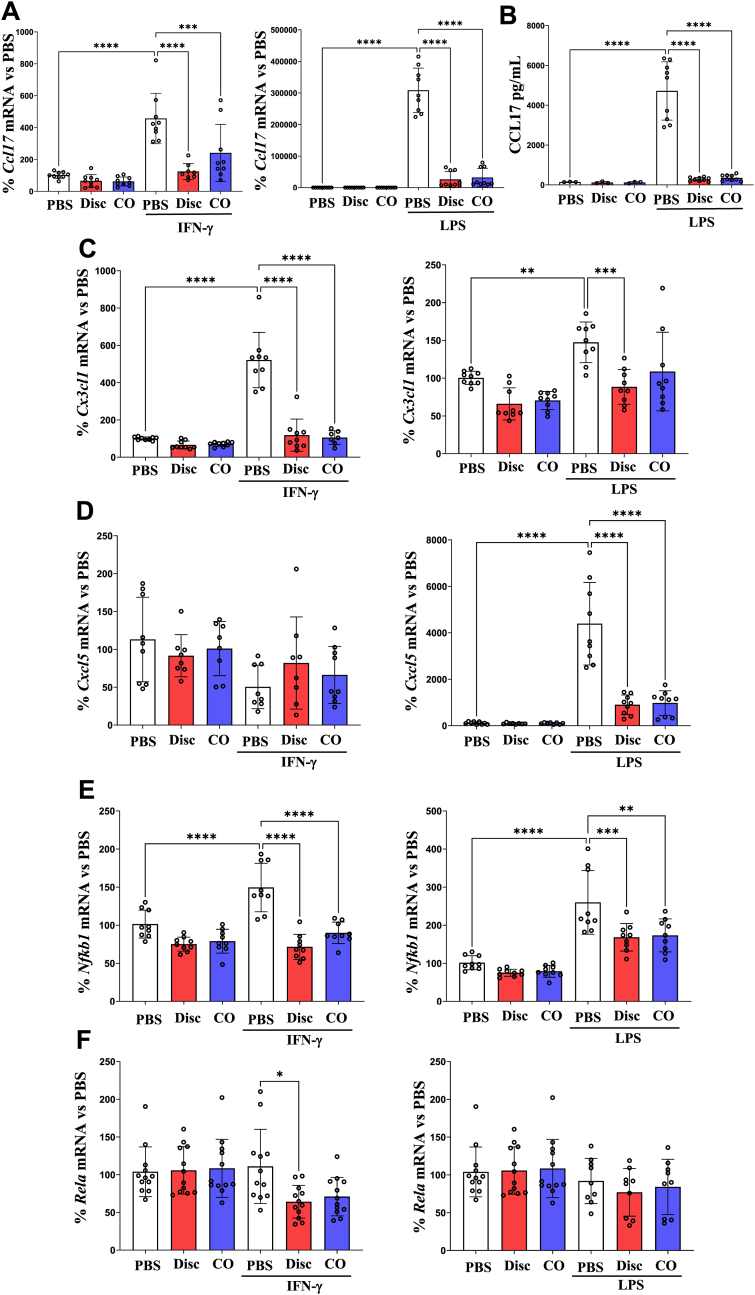
Por-NPs inhibit expression of inflammatory mediators in macrophages *in vitro.* (**A**) RT-qPCR measurement of *Ccl1*7 mRNA levels following incubation with discoidal (Disc) or CO-loaded (CO) Por-NPs and stimulation with IFN-γ (10 ng/mL) or LPS (10 ng/mL) and **(B)** ELISA measurement of secreted CCL17 protein in culture media. RT-qPCR assessment of **(C)***Cx3cl1,* (**D**) *Cxcl5,* (**E**) *Nfkb1* and (**F**) *Rela* mRNA following incubation with discoidal (Disc) or CO-loaded (CO) Por-NPs and stimulation with IFN-γ (10 ng/mL) or LPS (10 ng/mL) in iBMDMs (n = 8–9 biological replicates). Data expressed as Mean ± SD. ∗*P* < 0.05, ∗∗*P* < 0.01, ∗∗∗*P* < 0.001, ∗∗∗∗*P* < 0.0001 by one-way ANOVA with post-hoc Tukey's multiple comparisons.

In macrophages, Por-NPs inhibited expression of several additional inflammatory chemokines that are implicated in atherosclerosis progression. A significant decrease in *Ccl1*7 mRNA expression (IFN-γ: Disc, −73 %; CO, −47 %; *P* < 0.001/LPS: Disc, −92 %; CO, −90 %, *P* < 0.0001, Fig. 3A) and concomitant reduction in CCL17 protein in the cell culture media following LPS stimulation (Disc: 94 %; CO: 93 %, *P* < 0.0001, Fig. 3B).

There were also reductions observed in *Cx3cl1* (IFN-γ: Disc, −77 %; CO, −80 %; *P* < 0.0001/LPS: Disc, −40 %, *P* < 0.001, Fig. 3C) and *Cxcl5* (LPS: Disc, −80 %; CO, −78 %, *P* < 0.001, Fig. 3D) expression. Por-NPs led to a decrease in expression of *Nfkb1* (p50 subunit of NFκB) following stimulation with IFN-γ (Disc: 52 %; CO: 40 %, *P* < 0.0001) or LPS (Disc: 35 %; CO: 33 %, *P* < 0.01, Fig. 3E). Despite this, only the discoidal Por-NP significantly lowered expression of *Rela* (p65 subunit of NFκB) following IFN-γ stimulation (−42 %, *P* < 0.05, Fig. 3F)*.* There were no changes observed in other pro-inflammatory genes *Mif, Ccl8, Ccl7* or *Ccl3* (Fig. S4A–H).

### 3.3 Role of SR-BI and cholesterol efflux in the anti-inflammatory properties of Por-NPs in macrophages

We next determined whether the anti-inflammatory properties of Por-NPs in macrophages were mediated via SR-BI, the target receptor of R4F, *in vitro*.

**Fig. 4:**
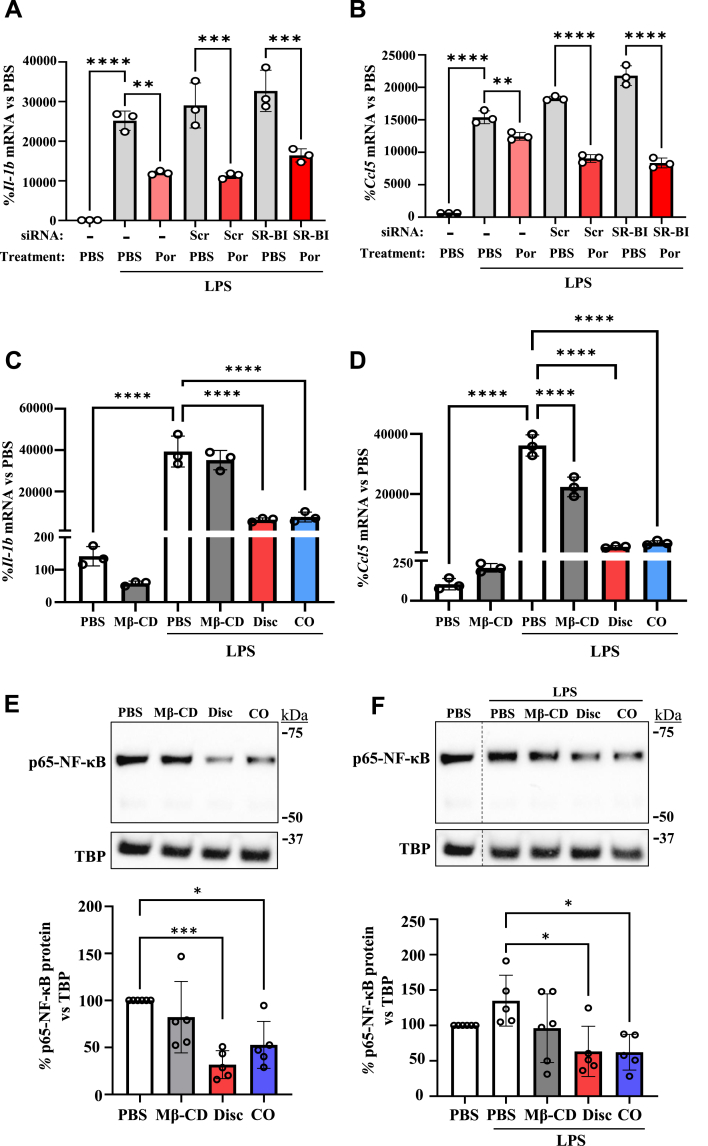
**Role of SR-B**I **and cholesterol efflux in the anti-inflammatory properties of Por-NPs in macrophages**. iBMDMs were transfected with SR-BI siRNA (100 nM) to knockdown SR-BI. RT-qPCR measurement of **(A)***Il1b* mRNA and **(B)***Ccl5* mRNA following incubation with discoidal Por-NPs and stimulation with LPS (10 ng/mL) (n = 3 biological replicates). iBMDMs were pre-incubated with PBS, methyl-β-cyclodextrin (MβCD, 4 % v/v), discoidal or CO-loaded Por-NPs (10 μg/mL) then stimulated with LPS (10 ng/mL) before RT-qPCR quantification of the mRNA levels of **(C)***Il1b* and **(D)***Ccl5*. The nuclear fractions of **(E)** unstimulated and **(F)** LPS-stimulated iBMDMs were isolated and nuclear p65-NFκB protein levels (∼65 kDa) were assessed by Western blotting (n = 5–6 biological replicates). TATA binding protein (TBP, 37 kDa) was used as the nuclear protein loading control. Data expressed as Mean ± SD. ∗*P* < 0.05, ∗∗*P* < 0.01, ∗∗∗*P* < 0.001, ∗∗∗∗*P* < 0.0001 by one-way ANOVA with post-hoc Tukey's multiple comparisons.

First, we confirmed that SR-BI protein was lower in SR-BI siRNA transfected cells than scrambled (Scr) siRNA controls (Supplemental Fig. S5A, −87°%, *P* < 0.0001). Despite SR-BI knockdown, significant reductions in *Il1b* and *Ccl5* mRNA levels were still observed following treatment with Por-NPs (Fig. 4A–B), suggesting the anti-inflammatory effects of Por-NPs are SR-BI-independent.

We next assessed the role of cholesterol efflux in the anti-inflammatory actions of Por-NPs using passive cholesterol acceptor (depletion) agent methyl-β-cyclodextrin (MβCD). MβCD removed 48 % of the loaded [^3^H]-cholesterol, confirming its efflux capabilities (Supplemental Fig. S5B, 8-fold, vs PBS control, *P* < 0.0001). This was greater than the cholesterol efflux induced by discoidal or CO-loaded Por-NPs. With LPS stimulation, *Il1b* mRNA levels were not different between the PBS and MβCD treatment groups (Fig. 4C), yet Por-NPs caused significant reductions in *Il1b* mRNA (Fig. 4C, Disc: 84 %; CO: 81 %, *P* < 0.0001). MβCD did, however, significantly decrease *Ccl5* mRNA levels (Figs. 3D, −38 %, p < 0.0001), compared to LPS stimulated controls, but Por-NPs caused a greater reduction in *Ccl5* mRNA levels (Fig. 4D, Disc: 89 %; CO: 84 %, *P* < 0.0001). These findings suggest inhibition of *Ccl5* but not *Il1b* by Por-NPs may, in part, be via increased cholesterol efflux.

NFκB is a pivotal inflammatory transcription factor that drives cytokine and chemokine expression. When activated, the p65 subunit translocates to the nucleus to upregulate inflammatory gene expression. We next determined changes in p65-NFκB protein in the nuclear fraction of iBMDMs. In non-stimulated iBMDMs, incubation with Por-NPs caused a reduction in nuclear p65-NFκB protein levels compared to the PBS control (Fig. 4E, Disc: 68 %, *P* < 0.0001; CO: 47 %, *P* < 0.05). There was no change in nuclear p65-NFκB protein in response to MβCD in non-stimulated iBMDMs. In LPS-stimulated iBMDMs, both discoidal and CO-loaded Por-NPs reduced nuclear p65-NFκB protein levels (Fig. 4F, Disc: 53 %, *P* < 0.0001; CO: 54 %, *P* < 0.05), compared to LPS control cells. This inhibitory effect was largely independent of cholesterol efflux as incubation with MβCD did not cause a significant reduction in nuclear p65 protein (−28 %, *P* = 0.17).

### 3.4 Por-NPs suppress components of the NLRP3 inflammasome

Por-NPs supressed NLRP3 inflammasome-associated cytokines IL-1β and IL-18. We next measured changes in key components of this pathway. Incubation of iBMDMs with Por-NPs significantly lowered *Nlrp3* mRNA levels (Supplemental Fig. S6A, Disc: 69 %, p < 0001; CO: 62 %, p < 0.001) compared to IFN-γ stimulated controls. No changes in *Nlrp3* mRNA levels were observed with Por-NP incubations following LPS stimulation (Supplemental Fig. S6B). Discoidal Por-NPs significantly reduced *Asc* mRNA levels in response to IFN-γ stimulation (Supplemental Fig. S6C, −36 %, *P* < 0.01). In iBMDMs stimulated with LPS, discoidal and CO-loaded Por-NPs reduced *Asc* mRNA levels as compared to the PBS non-stimulated controls (Supplemental Fig. S6D, Disc: 56 %, p < 0001; CO: 55 %, p < 0.001).

Overall, Por-NPs suppress the activation of the inflammatory transcription factor p65-NFκB, inflammatory cytokines and chemokines and components of the NLRP3 inflammasome, all of which are atheroprotective effects.

### 3.5 Detection of Por-NPs in murine plaques using multimodal imaging

**Fig. 5:**
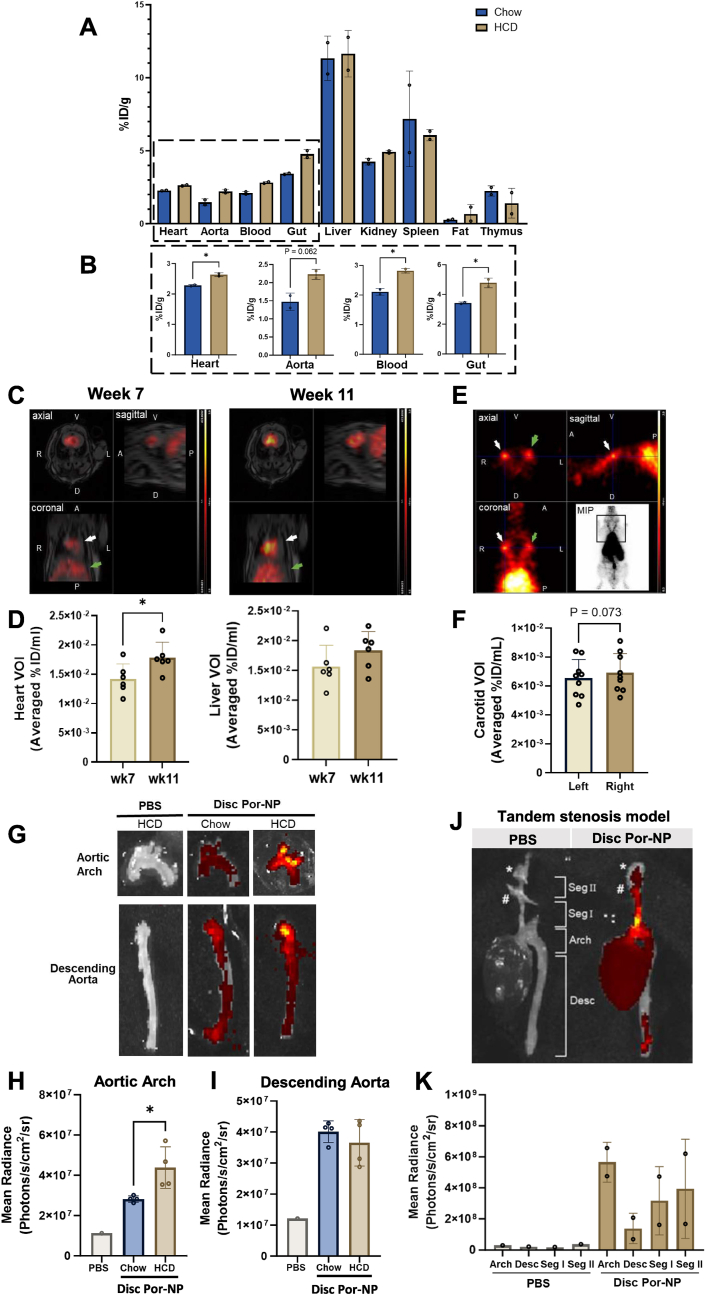
Biodistribution and multimodal imaging of Por-NPs in atherosclerotic mice. **(A)** γ-Counting of ^64^Cu-Por-NPs in organs from *Apoe*^*−/−*^ mice fed chow or HCD for 32 weeks (n = 2 animals/group). **(B)** Comparisons of γ-counts between chow and HCD-fed mouse organs. **(C)** Merged PET/MRI images of HCD fed *Apoe*^*−/−*^ mice at 7 and 11 weeks of HCD, 6 h after intravenous ^64^Cu-Por-NPs (White arrow, heart region. Green arrow, liver region). **(D)** VOI measurements of PET signal in heart and liver regions (n = 6 animals/group). ∗*P <* 0.05 by two-tailed paired *t*-test. **(E)** PET images at 6 weeks post-surgery (week 12 of HCD) in *Apoe*^*−/−*^ mice with tandem stenoses in the right carotid artery, 6 h post-intravenous ^64^Cu-Por-NPs. White and green arrows indicate right and left carotid arteries, respectively with **(F)** quantification of the PET activity in left and right carotid VOIs (n = 9 animals/group). **(G)** Representative IVIS images of excised aortic arch and descending aortas of PBS or Por-NP infused mice fed chow or HCD for 8 weeks with quantification of the fluorescence signal from aortic arch **(H)** and descending aorta **(I)** images (n = 1–4 animals/group), expressed as mean radiance (Photons/s/cm^2^/sr). ∗*P <* 0.05 by two-tailed unpaired *t*-test. (**J**) IVIS fluorescence images of *Apoe*^*−/−*^ mice with tandem stenoses in the right carotid with distal (∗) and proximal (#) sutures indicated, and Segments I & II (Seg I & II) shown and (**K**) quantified fluorescence (n = 1–2 animals/group). Data expressed as Mean ± SD. V, Ventral; D, Dorsal; R, Right; L, Left; A, Anterior; P, Posterior. ID, Injected dose. VOI, Volume of interest. MIP, Maximal Image Projection. (For interpretation of the references to colour in this figure legend, the reader is referred to the Web version of this article.)

*In vitro*, discoidal and CO-loaded Por-NPs were found to be taken up into iBMDM macrophages and elicit cholesterol efflux and anti-inflammatory effects equally. Discoidal Por-NPs were selected to be taken forward for testing *in vivo*. To determine the biodistribution of Por-NPs *in vivo*, we infused ^64^Cu-labelled Por-NPs into *Apoe*^*−/−*^ mice fed a chow or high cholesterol diet (HCD) for 32 weeks. *Apoe*^*−/−*^ mice fed HCD will develop more atherosclerosis than chow-fed mice over 32 weeks [21]. For chow and HCD mice, the highest uptake of ^64^Cu-labelled Por-NPs was in the liver (Fig. 5A). Uptake of ^64^Cu-Por-NPs was significantly higher in the heart of HCD-fed mice, where plaque accumulates in the sinus, compared to chow-fed mice (Fig. 5B, +15 %, *P* < 0.05). There was a non-significant trend for higher uptake of ^64^Cu-Por-NPs in the aortas of mice fed HCD compared to chow (Fig. 5B, +51 %, *P* = 0.062). We also found a significantly higher uptake of ^64^Cu-Por-NPs in whole blood (Fig. 5B, +34 %, *P* < 0.05) and gut (Fig. 5B, +39 %, *P* < 0.05) in HCD-fed mice compared to chow controls. Overall, this provides evidence for increased localization of Por-NPs in plaque regions in HCD-fed *Apoe*^−/−^ mice and in circulating blood cells that contribute to plaque development.

We next sought to determine whether ^64^Cu-labelled Por-NPs could be used to identify changes in plaque size longitudinally over time (Fig. 5C–D). PET/MRI imaging of *Apoe*^*−/−*^ mice fed a HCD demonstrated a quantifiable increase in PET signal in the heart VOI from week 7 to week 11 of HCD feeding (Fig. 5D, Week 7, 1.42x10^−2^ %ID/mL *vs.* Week 11, 1.741x10^−2^ %ID/mL, *P* < 0.05). There was no change in PET signal in the liver VOI over the same 4-week period of time (Fig. 5D, Week 7, 1.567x10^−2^ %ID/mL; Week 11, 1.864x10^−2^ %ID/mL). We next examined whether Por-NPs localized to carotid plaque in the murine tandem stenosis model of unstable atherosclerosis. ^64^Cu-Por-NPs were detected in the left and right carotid regions using PET imaging (Fig. 5E). When comparing left (non-surgical control) and right (tandem stenosis surgery) carotid regions, there was a non-significant increase in ^64^Cu-Por-NP signal in right carotid plaque (Fig. 5F, Left, 6.533x10^−3^ %ID/mL; Right, 6.9x10^−3^ %ID/mL; P = 0.073), compared to the left carotid.

We next explored the fluorescence imaging capabilities of Por-NPs and ability to track to plaque in HCD-fed *Apoe*^*−/−*^ mice. Using IVIS fluorescence imaging, excised aortic arches and descending aortas were compared following infusion of PBS or Por-NPs (Fig. 5G). HCD-fed mice elicited a stronger fluorescence signal in the arch region, where plaque develops, than chow-fed Por-NP controls (Fig. 5H, +55 %, *P* < 0.05). In contrast, there were no changes in fluorescence detected between HCD and chow-fed controls in the descending aorta (Fig. 5I), a site with little plaque after 8 weeks HCD. Por-NP fluorescence was also quantified in the liver, kidney, spleen and lung (Supplemental Fig. S7A). Quantification of the fluorescence signal revealed that Por-NP fluorescence was greatest in the liver of HCD fed mice compared to chow-fed control livers (Fig. S7B, 2-fold, *P* < 0.0001). The fluorescence signal from the livers of HCD-fed mice was significantly higher than the other organs including the kidney, spleen and lungs (Fig. S7B, ∼10-fold, *P* < 0.0001 for all). In animals subjected to carotid tandem stenosis surgery, IVIS fluorescence imaging was also conducted *ex vivo* on the vascular tree (Fig. 5J). Fluorescence was quantified from different regions including aortic arch, descending aorta, and tandem stenosis segments I and II, with fluorescence detected in the Por-NP but not PBS group (Fig. 5K). The fluorescence signal was highest in the aortic arch, compared to the descending aorta, and was also detected in segment I and II of the carotid arteries with tandem stenoses. The importance of the R4F peptide in Por-NPs for plaque uptake *in vivo* was examined. We compared Por-NPs to porphyrin-lipid micelles (Por-micelle) that had no peptide (Supplemental Fig. S8A-B) and found no changes in plaque uptake, as measured *ex vivo* by IVIS fluorescence imaging, in the descending aorta of mice treated with either Por-NPs or Por-micelles (Supplemental Fig. S8D-F).

**Fig. 6:**
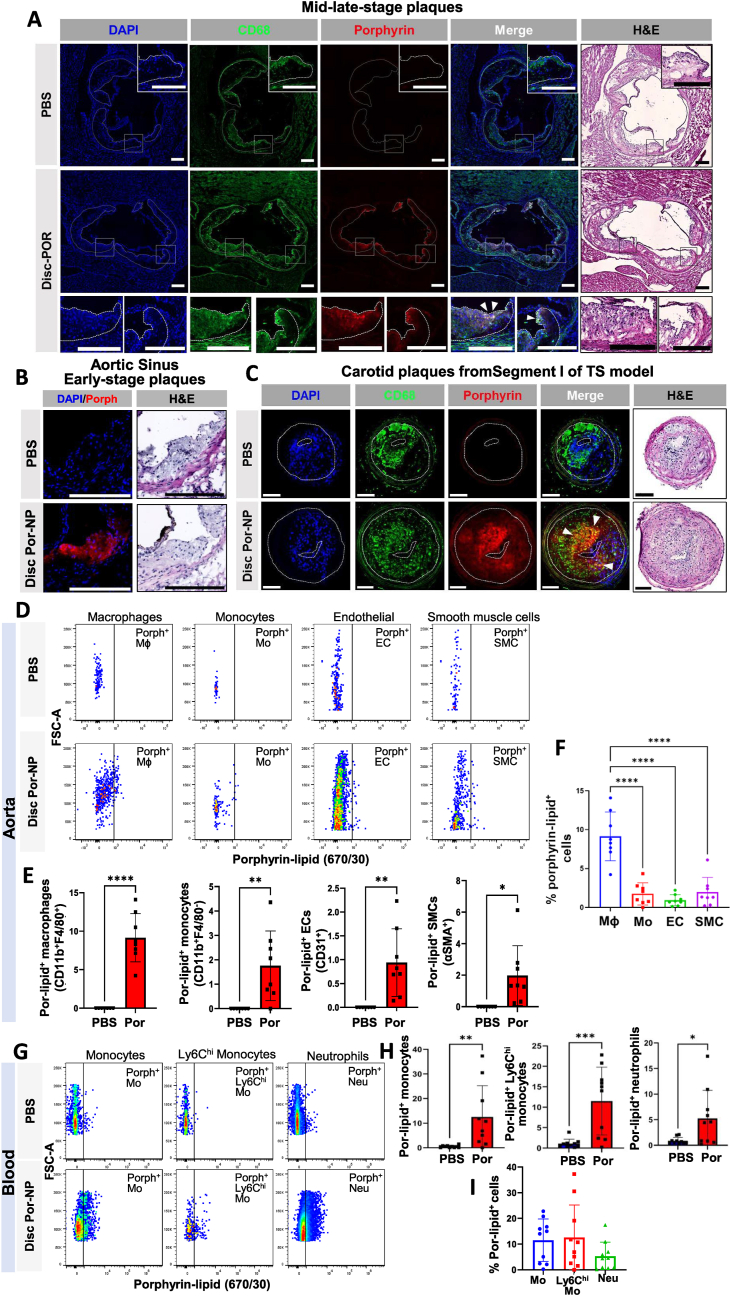
**Por-NP fluorescence detected in plaques, aortas and circulating cells *in vivo.*** (A) Fluorescence microscopy of mid-stage aortic sinus plaque sections from *Apoe*^−/−^ mice infused with PBS or Por-NPs showing nuclei (DAPI, blue), CD68^+^ macrophages (FITC, green), porphyrin-lipid (Cy5-filter, red) with colocalization (white, indicated by white arrows) and adjacent H&E images. Scale bar: 200 μm. (B) Fluorescence microscopy of early-stage aortic sinus plaque sections from *Apoe*^−/−^ mice infused with PBS or Por-NP showing nuclei (DAPI, blue) and porphyrin-lipid (red) and adjacent H&E images. Scale bar: 200 μm. (C) Fluorescence microscopy of tandem stenosis carotid artery plaque (Segment I) sections, from mice infused with PBS or Por-NP, showing nuclei (DAPI, blue), CD68^+^ macrophages (green), porphyrin lipid (red) with colocalization (yellow, indicated by white arrows) and adjacent H&E images. Scale bar: 100 μm. (D) Descending thoracic aortas analyzed by flow cytometry for porphyrin-lipid fluorescence in: (E) macrophages (MΦ), monocytes (Mo), endothelial cells (ECs) and smooth muscle cells (SMCs) (n = 6–8 animals/group). (F) Comparison of porphyrin-lipid uptake into aortic cell types. (G) Circulating cells in blood assessed by flow cytometry for porphyrin-lipid fluorescence in: (H) monocytes, Ly6C^hi^ activated monocytes and neutrophils. (I) Comparison of porphyrin-lipid uptake into circulating cells (n = 8 animals/group). Data expressed as Mean ± SD. ∗*P* < 0.05, ∗∗*P* < 0.01, ∗∗∗*P* < 0.001, ∗∗∗∗*P* < 0.0001 vs PBS by two-tailed unpaired *t*-test or ∗∗∗∗*P* < 0.0001 by one-way ANOVA with post-hoc Tukey's multiple comparisons. Por: Por-NP. Por-lipid: Porphyrin-lipid. (For interpretation of the references to colour in this figure legend, the reader is referred to the Web version of this article.)

Next, using fluorescence microscopy we showed that Por-NP uptake localizes to plaques in the aortic sinus and in Segment I of carotid arteries subjected to tandem stenosis surgery. In mid-stage plaques of the aortic sinus, porphyrin-lipid fluorescence was localized in the shoulder and luminal-facing regions (Fig. 6A). We found porphyrin-lipid fluorescence (red) co-localized (in white) with regions of CD68^+^ macrophages (green) in the plaque (Fig. 6A, marked by white arrows). In early-stage aortic sinus plaque, porphyrin-lipid fluorescence was present in the majority of the plaque in Por-NP infused but not PBS-treated mice (Fig. 6B). Moreover, porphyrin-lipid fluorescence (red) was present in plaque of segment I from carotid arteries subjected to tandem stenosis surgery in the Por-NP treated mice and was co-localized (yellow) with CD68^+^ macrophages (green) (Fig. 6C, marked by white arrows).

Using flow cytometry, we detected porphyrin-lipid positive macrophages, monocytes, endothelial cells and smooth muscle cells in digested aortas excised from *Apoe*^−/−^ mice infused with Por-NPs, which were not present in PBS controls (Fig. 6D–E). In *Apoe*^*−/−*^ mice infused with Por-NPs, the highest proportion of porphyrin-lipid positive cells were in the macrophage population (9 %), with comparatively small amounts in the monocyte (1.8 %), endothelial (0.09 %) and smooth muscle (2 %) cell populations (Fig. 6F, *P* < 0.0001). In blood, porphyrin-lipid fluorescence was detected in circulating monocytes (Fig. 6G–H, 11.5 %), activated monocytes (Fig. 6G–H, 12.5 %) and neutrophils (Fig. 6, Fig. 5.2 %). There were no significant differences in Por-NP uptake between these circulating cell populations (Fig. 6I).

### 3.6 Por-NPs exhibit anti-atherosclerotic properties *in vivo*

**Fig. 7:**
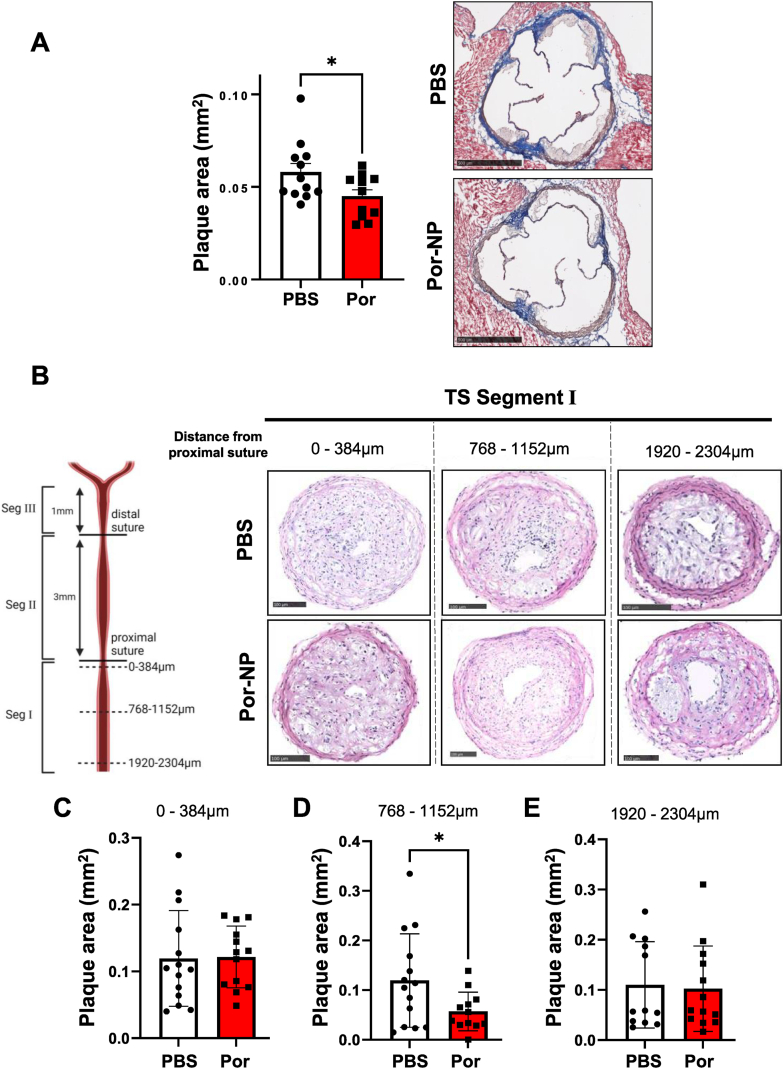
**Por-NPs reduce atherosclerotic plaque development**. (**A)***Apoe*^−/−^ mice were fed HCD for 6 weeks (early-stage plaque) and received PBS or Por-NP intraperitoneally on alternate days. Three sections spanning the aortic sinus were stained with Masson's Trichrome. Plaque area was calculated and averaged across sections (mm^2^, n = 12 animals/group). Right: representative aortic sinus sections. Scale bar: 500 μm. **(B)** After 6 weeks of HCD, *Apoe*^−/−^ mice received tandem stenosis surgery, then received PBS or Por-NPs intraperitoneally on alternate days for 7 weeks. **Left:** Sectioned regions of tandem stenosis segment I in the right carotid artery. Plaque area measured in three regions corresponding to 0–384 μm, 768–1152 μm, and 1920–2304 μm from the proximal suture respectively. **Right:** Representative images of H&E-stained tandem stenosis segment I sections within the three regions. Scale bar: 100 μm. Plaque area in tandem stenosis segment I quantified in regions: **(C)** 0–384 μm, **(D)** 768–1152 μm and **(E)** 1920–2304 μm. (n = 12–14 animals/group). Data expressed as mean ± SD. ∗*P* < 0.05 vs PBS control by two-tailed unpaired *t*-test. TS, Tandem Stenosis. Por: Por-NP. Seg: Segment.

We next tested the therapeutic effects of Por-NPs *in vivo* in early-stage plaque and unstable atherosclerosis models (Supplemental Fig. S9). For the early-stage plaque model, in 4-week-old *Apoe*^−/−^ mice, after 6 weeks of treatment, analysis of plaque in the aortic sinus revealed that Por-NP infused mice had significantly smaller plaques (Figs. 7A, −23 %, *P* < 0.05) than PBS controls. There were, however, no changes in the % of plaque CD68^+^ macrophages and α-SMA^+^ smooth muscle cells between treatment groups (Supplemental Fig. S10A-B). In the tandem stenosis unstable plaque model, *Apoe*^−/−^ mice were treated for 7 weeks following surgery. H&E-stained sections were analyzed systematically across three regions spanning tandem stenosis Segment I [18] (Fig. 7B). At the mid-point region of Segment I (768–1152 μm), Por-NP-treated mice developed significantly smaller plaques, compared to PBS controls (Figs. 7D, −52 %, *P* < 0.05). No differences in plaque size were observed in the outer regions of Segment I (0–383, Fig. 7C and 1920–2304 μm, Fig. 7D).

There were no changes in the percent area of plaque CD68^+^ macrophages, Oil Red O lipid, α-SMA^+^ smooth muscle cells or TER-119^+^ erythrocytes between groups (Supplemental Fig. S10C-G). However, there was an observed decrease in percent area of collagen content (Fig. S10H, −48 %, *P* < 0.05). Additionally, the percent extracellular lipid area of the plaque also was not significantly different between treatment groups (Fig S10I).

**Fig. 8:**
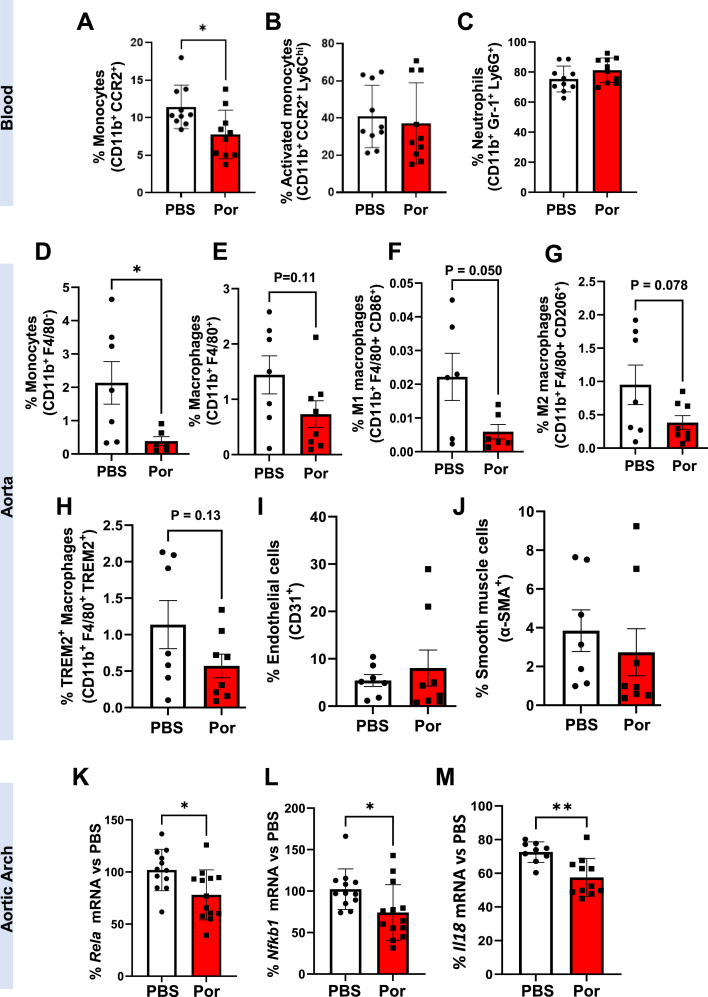
**Por-NPs reduce circulating monocyte and aortic macrophages and inhibit aortic *Rela in vivo.*** Circulating **(A)** monocytes, **(B)** activated monocytes and **(C)** neutrophils were measured and quantified in blood using flow cytometry in mice fed a HCD for 6 weeks (n = 10 animals/group). Aortic **(D)** monocytes, **(E)** macrophages, **(F)** M1 macrophages, **(G)** M2 macrophages, **(H)** TREM2^+^ foam cell macrophages, **(I)** endothelial cells and **(J)** and smooth muscle cells were measured and quantified in the descending aorta using flow cytometry in *Apoe*^−/−^ mice fed a HCD for 13 weeks (n = 7–8 animals/group). qPCR of RNA extracted from the aortic arch of mice fed a HCD for 13 weeks to determine mRNA levels of: **(K)***Il1b,***(L)***Ccl5,***(M)***Ccl2* and **(N)***Rela* (n = 12–14 animals/group). Data expressed as mean ± SD. ∗*P* < 0.05 vs PBS control by two-tailed unpaired *t*-test. Por: Por-NP.

We next examined changes in circulating and aortic cells by flow cytometry. In blood, we found a significant reduction in circulating monocytes in mice treated with Por-NPs (Figs. 8A, −32 %, *P* < 0.05). However, no changes were observed in activated monocytes (Fig. 8B) or neutrophils (Fig. 8C). Analyses of aortas from treated mice found a significant reduction in monocyte content (Figs. 8D, −81 %, *P* < 0.05) in mice infused with Por-NPs, when compared to PBS controls. There were non-significant reductions in aortic macrophages (Figs. 8E, −49 %, *P* = 0.11) and M1 (Figs. 8F, −73 %, *P* = 0.050) and M2 (Figs. 8G, −77 %, *P* = 0.078) macrophage phenotypes, and TREM2^+^ foam cells (Figs. 8H, −50 %, *P* = 0.13) in Por-NP treated mice. No changes were observed in aortic endothelial and smooth muscle cell numbers between groups (Fig. 8I–J).

Whilst no differences were observed in aortic arch mRNA levels of inflammatory genes *Il1b*, *Ccl5, Ccl2, Ccl17, Ccl8, Ccl7, Ccl3, Cx3cl1* or *Mif* (Fig. S11A-H, J), there was a significant reduction in aortic arch *Rela* (Figs. 8K, −26 %, *P* < 0.05)*, Nfkb1* (Fig.s. 8L, −27 %, *P* < 0.05) and *Il18* (Fig.s. 8M, −21 %, *P <* 0.01) mRNA in Por-NP treated mice compared to PBS controls. Mice given Por-NPs had significantly higher aortic arch *Cxcl5* (Fig. S11I, 42 %, *P* < 0.05). In plasma, Por-NP mice had a trend for lower CCL17 (Fig. S11K, −12 %, *P* = 0.06) and elevated CCL5 (Fig. S11L, 71 %, *P <* 0.01).

In both the early plaque and unstable plaque models, there were no changes in plasma total cholesterol, LDL cholesterol or HDL cholesterol between treatment groups (Supplemental Table S5 and Supplemental Table S6). In the unstable plaque model, there was a significant reduction in plasma triglyceride concentrations (Supplemental Table S6, -22.9 %, *P* < 0.01) in Por-NP treated mice.

## 4 Discussion

Nanotechnologies have emerged as highly promising novel CVD treatment and diagnostic strategies with macrophages presenting as desirable cellular targets [6]. A significant advantage of nanoparticles is they can be synthesized to combine therapeutic and diagnostic components into a single theranostic nanoscale platform [7]. This study tested Por-NPs for their ability to detect and suppress atherosclerosis. We report the following important findings: 1) Por-NPs are internalized by macrophages, which can be visualized by fluorescence microscopy and detected by flow cytometry; 2) Por-NPs exhibit anti-atherosclerotic effects *in vitro* in macrophages and promote cholesterol efflux and inhibit inflammation; 3) Por-NPs localize within plaques *in vivo* and can be detected using PET and fluorescence imaging; and 4) Por-NP uptake in plaque is primarily by macrophages. 5) Infusions of Por-NPs reduce early-stage and unstable plaque development, and decrease circulating and aortic monocytes. Our findings demonstrate the theranostic potential of Por-NPs for atherosclerosis.

Por-NPs contain peptide R4F, which enables interaction with the SR-BI receptor [15]. SR-BI is expressed and functionally utilized by macrophages to facilitate both efflux and influx of cholesterol, and mediates activation of downstream signaling pathways [17]. Other R4F nanoparticles are reported to interact with SR-BI in Chinese hamster ovary cells [15]. Here we observed Por-NP uptake by macrophages (iBMDMs), consistent with other nanoparticles containing R4F [[22], [23], [24]]. To our knowledge, this is the first time R4F-containing Por-NPs have been shown to be internalized by macrophages, visualized using the fluorescent properties of porphyrin-lipid (Fig. 1B). We also confirmed that this uptake is most likely facilitated by SR-BI, as we found that uptake was significantly increased by the presence of SR-BI (Fig S3). However, Por-micelles (without R4F) were taken up in comparable way to Por-NPs (with R4F) into atherosclerotic plaque *in vivo* (Fig S8). This may be because the endothelium overlying plaques has greater permeability, facilitating passive nanoparticle entry. Furthermore, macrophages, as professional scavengers, internalise nanoparticles through multiple receptors and endocytic pathways, independently of SR-B1.

Using PET and fluorescence imaging modalities we found ^64^Cu-Por-NPs tracked to atherosclerotic plaque *in vivo* (Fig. 5, Fig. 6). We also found the ^64^Cu-Por-NP signal was sensitive enough to detect measurable increases in plaque in the heart region. In longitudinal measures in *Apoe*^−/−^ mice, serial PET imaging showed an increase in PET signal in the heart region over a period of 4 weeks on HCD. There were, however, no changes noted in ^64^Cu-Por-NP signal between the right (surgical) and the left (non-surgical) carotid artery in the tandem stenosis model. The resolution of PET was likely insufficient to detect changes in ^64^Cu-Por-NPs signal between carotid arteries. The carotid artery is very small as is the plaque within it. Furthermore, the left carotid artery will develop some stable plaque due to the HCD. A unique feature of the Por-NP design is that the integrated porphyrin-lipid does not require the conjugation of additional fluorophores [10,11,25]. Using IVIS imaging, we could therefore also detect higher porphyrin-lipid fluorescence signal in the aortic arch of HCD-fed *Apoe*^*−/−*^ mice, compared to chow fed controls with no plaque. This indicates specific uptake in a known region of plaque deposition [26], supporting the PET observations. The non-specific distribution in other organs, as detected by *ex vivo* fluorescence imaging, is likely attributed to the presence of SR-BI which is also expressed in the liver, the site of the highest uptake (Supplemental Fig. S7). This distribution is consistent with previous studies of HDL-like NPs [11,27]. Consistent with the observations in the aortic arch, we observed porphyrin-lipid fluorescence in tissue sections of aortic sinus plaque and unstable plaque, co-localized with CD68^+^ macrophages, by fluorescence microscopy. The plaque targeting capabilities of Por-NPs were further demonstrated in biodistribution studies that showed higher activity of ^64^Cu-Por-NPs in the heart and aorta of HCD-fed mice with established plaque than chow-fed mice with little-to-no plaque. Flow cytometry revealed porphyrin-lipid uptake was highest in aortic macrophages and was detected in circulating monocytes. Circulating monocytes infiltrate and differentiate into macrophages within atherosclerotic lesions [28], suggesting porphyrin-lipid fluorescence is retained in this process. In summary, Por-NPs are taken up preferentially by aortic macrophages and are detected in atherosclerotic plaque, our target site of interest. This makes them desirable agents for detecting the presence of plaque, including early-stage plaque.

The present study found Por-NPs inhibited macrophage expression of pro-inflammatory cytokines IL-1β and IL18, and the chemokine CCL5. IL-1β and IL18 have a key role in atherogenesis [[29], [30], [31]] and their release can result from NLRP3 inflammasome activation [32,33] (Fig. 2). Por-NPs inhibited the expression of NLRP3 inflammasome components, *Nlrp3* and *Asc*, which may in part explain the reduction in IL-1β and IL18. Interestingly, knockdown of SR-BI did not affect the anti-inflammatory actions of Por-NPs, given that SR-BI is the target receptor of R4F (Fig. 4). Using the passive cholesterol efflux agent, methyl-β-cyclodextrin (MβCD), we found that whilst cholesterol efflux had no effect on the inhibitory effects of Por-NPs on *Il1b*, MβCD caused a significant reduction in *Ccl5*. This suggests that cholesterol efflux has some involvement in mediating the inhibitory effects of Por-NPs on *Ccl5*, but only in part, with Por-NPs causing a more substantial reduction in *Ccl5* than MβCD.

Additionally, Por-NPs were able to inhibit the expression of other chemokines *Ccl17*, *Cx3cl1* and *Cxcl5,* also inhibiting the release of CCL17 protein from macrophages *in vitro* (Fig. 3). CCL17 has been previously shown to drive atherosclerosis through suppression of regulatory T-cell functions [34,35] and *Cx3cl1* is an established as a pro-atherogenic chemokine [36]. In contrast, *Cxcl5* expression increased in animals treated with Por-NPs in the aortic arch *in vivo.* However, CXCL5 has been shown previously to reduce foam cell formation in atherosclerosis [37], so upregulation might lead to an overall anti-atherogenic effect *in vivo*.

Por-NPs suppressed p65-NF-κB activation *in vitro* in iBMDMs. Accordingly, Por-NPs also inhibited *Rela* and *Nfkb1* expression*,* genes that transcribe subunits p65 and p50 of NF-κB respectively*,* in aortic arches *in vivo*. NF-κB is the pivotal transcription factor that controls the expression of inflammatory cytokines and chemokines, and also primes NLRP3 inflammasome assembly [33]. The suppression of p65-NF-κB activation by Por-NPs supports the inhibition of IL-1β, *Il18* and CCL5, as well as NLRP3 inflammasome components *Nlrp3* and *Asc*. Using MβCD, the inhibitory effect of Por-NPs on NF-κB was largely independent of cholesterol efflux. Overall, Por-NPs exhibit anti-inflammatory properties *in vitro* in macrophages and *in vivo* in the aortic arch through inhibition of NF-κB.

We found Por-NPs were highly efficient cholesterol efflux acceptors (Fig. 2). By reducing foam cell formation, cholesterol efflux is associated with lower incidence of CAD [38,39]. The anti-inflammatory and cholesterol efflux properties of Por-NPs suggest they will have atheroprotective effects. Consistent with this, we found Por-NPs reduced plaque area *in vivo* in early-stage plaque and tandem stenosis unstable atherosclerosis models. Furthermore, we observed reductions in circulating and aortic monocytes. These atheroprotective properties occurred independently of changes in plasma cholesterol, highlighting their potential to add benefit on top of lipid-lowering therapies. Despite the significant changes observed in cholesterol efflux in macrophages *in vitro*, we did not see changes in the lipid content of plaques as measured by Oil Red O. This may be because this histological measure reflects the net effect of multiple cell types, and the efflux component is only a very small proportion of total plaque lipid.

Despite the novelty of this project some limitations exist. Due to their importance in atherosclerosis, our *in vitro* studies focused on testing macrophages. SR-BI, the target of Por-NPs, is expressed on macrophages but also on other vascular cell types in plaque. Whilst it is anticipated that Por-NPs would elicit beneficial effects in other cell types, this remains unexplored. R4F is designed to interact with SR-BI, however, there may be other receptor mediated uptake mechanisms at play beyond SR-BI interactions.

Future clinical applications of Por-NP technology might their utility for early detection, in parallel with established anti-atherosclerotic therapies. Practically the fluorescence detection system would be most suited to intravascular imaging, as the penetration depth of fluorescence imaging cannot reach the coronary arteries of the heart. This has been explored recently, with Por-NPs imaged using a dual fluorescence/OCT intravascular probe in atherosclerotic plaque of *Apoe*^−/−^ [40]. In addition, Por-NPs can be utilized as a plaque macrophage PET tracer for plaque imaging. Ultimately Por-NPs could also act simultaneously as a treatment to inhibit atherosclerosis, with the potential to load cargo within the core that extends their therapeutic capacity for targeted drug delivery to the plaque site. Future work in large animal models is needed to further explore the clinical application of Por-NPs in cardiovascular medicine.

**Fig. 9:**
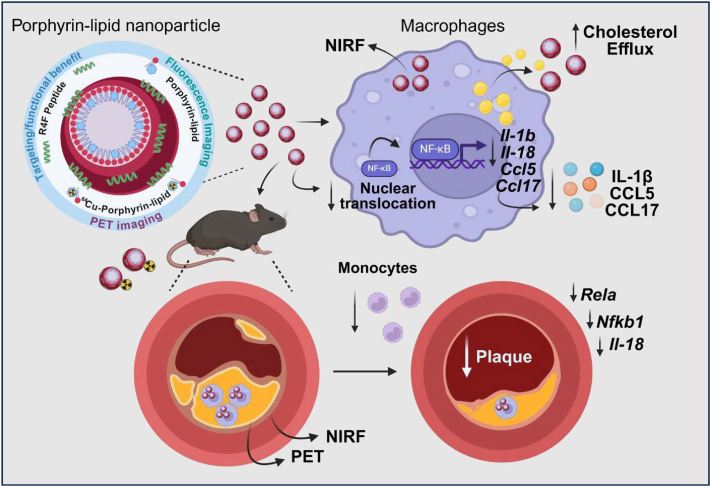
Theranostic properties of porphyrin-lipid nanoparticles *in vitro* and *in vivo.* Porphyrin-lipid nanoparticles (Por-NPs) are taken up by macrophages *in vitro* and emit near infrared fluorescence (NIRF) that can be detected by fluorescence microscopy and flow cytometry. Por-NPs also elicit atheroprotective effects in macrophages; enhancing cholesterol efflux and supressing inflammatory mediators IL-1β, CCL5 and CCL17 via inhibition of the translocation of p65-NF-κB to the nucleus. *In vivo*, infused Por-NPs track to atherosclerotic plaques, colocalise with macrophages, and emit NIRF that can be detected by fluorescence IVIS imaging, confocal microscopy and flow cytometry. Por-NPs can be radiolabelled and infused Copper-64 (^64^Cu)-Por-NPs enable serial positron emission tomography (PET) imaging of plaques and could detect increases in plaque size over time. Por-NPs reduce the development of atherosclerotic plaques, reduce circulating and aortic monocytes and suppress aortic arch *Rela* and *Nfkb1*, that transcribe NF-κB subunits p65 and p50. In summary, Por-NPs are novel nanoscale theranostics for atherosclerotic cardiovascular disease.

In conclusion, Por-NPs exhibit diagnostic and therapeutic properties in atherosclerotic CVD (Fig. 9). *In vitro* Por-NPs are internalized by macrophages and promote cholesterol efflux. Por-NPs also elicit anti-inflammatory properties via suppression of p65-NF-κB. *In vivo*, Por-NPs localize to plaques and plaque macrophages as detected via fluorescence, using the intrinsic fluorescent properties of porphyrin-lipid. ^64^Cu-labelled Por-NPs longitudinally detect plaque growth in hearts using non-invasive PET imaging. Finally, Por-NPs reduce plaque development in early-stage and unstable tandem stenosis models of atherosclerosis. In parallel we observed reductions in circulating monocytes and aortic arch *Rela/Nfkb1,* genes that transcribe NF-κB – both potential mechanisms to explain the reduction in atherosclerosis. This is the first characterization of the theranostic properties of Por-NPs in atherosclerosis. Our findings have significant implications for the use of porphyrin-lipid nanoparticles that simultaneously improve diagnosis via multiple imaging modalities and prevent plaque development.

## CRediT authorship contribution statement

**Victoria A. Nankivell:** Writing – review & editing, Writing – original draft, Visualization, Validation, Methodology, Investigation, Formal analysis, Data curation, Conceptualization. **Lauren Sandeman:** Methodology, Investigation, Data curation. **Liam Stretton:** Writing – original draft, Visualization, Methodology, Investigation, Formal analysis, Data curation. **Achini K. Vidanapathirana:** Supervision, Methodology, Investigation, Data curation, Conceptualization. **Maneesha A. Rajora:** Resources, Methodology, Investigation, Formal analysis, Data curation, Conceptualization. **Juan Chen:** Validation, Resources, Methodology, Investigation, Formal analysis, Data curation. **William Tieu:** Visualization, Resources, Methodology, Investigation, Data curation. **Hanyi Weng:** Resources, Methodology, Data curation. **Maaike Kockx:** Resources. **Leonard Kritharides:** Resources. **Peter J. Psaltis:** Writing – review & editing. **Joanne T.M. Tan:** Writing – review & editing, Writing – original draft, Supervision, Investigation, Formal analysis. **Yung-Chih Chen:** Writing – review & editing, Methodology, Formal analysis, Data curation, Conceptualization. **Karlheinz Peter:** Writing – review & editing, Writing – original draft, Supervision, Methodology. **Gang Zheng:** Writing – review & editing, Writing – original draft, Supervision, Resources, Methodology, Funding acquisition, Data curation, Conceptualization. **Christina A. Bursill:** Writing – original draft, Visualization, Validation, Supervision, Software, Resources, Project administration, Methodology, Investigation, Funding acquisition, Formal analysis, Data curation, Conceptualization.

## Conflict of interests

The authors have no conflicts to declare.

## Data availability statement

The data of this research is available for sharing.

## Declaration of competing interest

The authors declare that they have no known competing financial interests or personal relationships that could have appeared to influence the work reported in this paper.

## Data Availability

Data will be made available on request.

## References

[1] World Health Organisation (2021 11 June 2021). Cardiovascular diseases (CVDs). https://www.who.int/en/news-room/fact-sheets/detail/cardiovascular-diseases.

[2] Fernández-Friera L., Fuster V., López-Melgar B., Oliva B., García-Ruiz J.M., Mendiguren J., Bueno H., Pocock S., Ibáñez B., Fernández-Ortiz A., Sanz J. (2017). Normal LDL-cholesterol levels are associated with subclinical atherosclerosis in the absence of risk factors. J. Am. Coll. Cardiol..

[3] Nankivell V., Vidanapathirana A.K., Hoogendoorn A., Tan J.T.M., Verjans J., Psaltis P.J., Hutchinson M.R., Gibson B.C., Lu Y., Goldys E., Zheng G., Bursill C.A. (2024). Targeting macrophages with multifunctional nanoparticles to detect and prevent atherosclerotic cardiovascular disease. Cardiovasc. Res..

[4] Duivenvoorden R., Tang J., Cormode D.P., Mieszawska A.J., Izquierdo-Garcia D., Ozcan C., Otten M.J., Zaidi N., Lobatto M.E., van Rijs S.M., Priem B., Kuan E.L., Martel C., Hewing B., Sager H., Nahrendorf M., Randolph G.J., Stroes E.S., Fuster V., Fisher E.A., Fayad Z.A., Mulder W.J. (2014). A statin-loaded reconstituted high-density lipoprotein nanoparticle inhibits atherosclerotic plaque inflammation. Nat. Commun..

[5] Flores A.M., Hosseini-Nassab N., Jarr K.-U., Ye J., Zhu X., Wirka R., Koh A.L., Tsantilas P., Wang Y., Nanda V., Kojima Y., Zeng Y., Lotfi M., Sinclair R., Weissman I.L., Ingelsson E., Smith B.R., Leeper N.J. (2020). Pro-efferocytic nanoparticles are specifically taken up by lesional macrophages and prevent atherosclerosis. Nat. Nanotechnol..

[6] Chen W., Schilperoort M., Cao Y., Shi J., Tabas I., Tao W. (2021). Macrophage-targeted nanomedicine for the diagnosis and treatment of atherosclerosis. Nat. Rev. Cardiol..

[7] Zhang Y., Koradia A., Kamato D., Popat A., Little P.J., Ta H.T. (2019). Treatment of atherosclerotic plaque: perspectives on theranostics.

[8] Rajora M.A., Lou J.W.H., Zheng G. (2017). Advancing porphyrin's biomedical utility via supramolecular chemistry. Chem. Soc. Rev..

[9] Liu T.W., MacDonald T.D., Shi J., Wilson B.C., Zheng G. (2012). Intrinsically Copper-64-Labeled organic nanoparticles as radiotracers. Angew. Chem. Int. Ed..

[10] Lovell J.F., Jin C.S., Huynh E., Jin H., Kim C., Rubinstein J.L., Chan W.C.W., Cao W., Wang L.V., Zheng G. (2011). Porphysome nanovesicles generated by porphyrin bilayers for use as multimodal biophotonic contrast agents. Nat. Mater..

[11] Cui L., Lin Q., Jin C.S., Jiang W., Huang H., Ding L., Muhanna N., Irish J.C., Wang F., Chen J., Zheng G. (2015). A PEGylation-Free biomimetic porphyrin nanoplatform for personalized cancer theranostics. ACS Nano.

[12] Muhanna N., Chan H.H.L., Townson J.L., Jin C.S., Ding L., Valic M.S., Douglas C.M., MacLaughlin C.M., Chen J., Zheng G., Irish J.C. (2020). Photodynamic therapy enables tumor-specific ablation in preclinical models of thyroid cancer. Endocr. Relat. Cancer.

[13] Muhanna N., Cui L., Chan H., Burgess L., Jin C.S., MacDonald T.D., Huynh E., Wang F., Chen J., Irish J.C., Zheng G. (2016). Multimodal image-guided surgical and photodynamic interventions in head and neck cancer: from primary tumor to metastatic drainage. Clin. Cancer Res..

[14] Ujiie H., Ding L., Fan R., Kato T., Lee D., Fujino K., Kinoshita T., Lee C.Y., Waddell T.K., Keshavjee S., Wilson B.C., Zheng G., Chen J., Yasufuku K. (2019). Porphyrin–high-density lipoprotein: a novel photosensitizing nanoparticle for lung cancer therapy. Ann. Thorac. Surg..

[15] Lin Q., Chen J., Ng K.K., Cao W., Zhang Z., Zheng G. (2014). Imaging the cytosolic drug delivery mechanism of HDL-Like nanoparticles. Pharm. Res..

[16] Shen W.-J., Azhar S., Kraemer F.B. (2018). SR-B1: a unique multifunctional receptor for cholesterol influx and efflux. Annu. Rev. Physiol..

[17] Linton M.F., Tao H., Linton E.F., Yancey P.G. (2017). SR-BI: a multifunctional receptor in cholesterol homeostasis and atherosclerosis. Trends in endocrinology and metabolism: TEM (Trends Endocrinol. Metab.).

[18] Chen Y.C., Bui A.V., Diesch J., Manasseh R., Hausding C., Rivera J., Haviv I., Agrotis A., Htun N.M., Jowett J., Hagemeyer C.E., Hannan R.D., Bobik A., Peter K. (2013). A novel mouse model of atherosclerotic plaque instability for drug testing and mechanistic/therapeutic discoveries using gene and microRNA expression profiling. Circ. Res..

[19] Rajora M.A., Ding L., Valic M., Jiang W., Overchuk M., Chen J., Zheng G. (2017). Tailored Theranostic apolipoprotein E3 porphyrin-lipid nanoparticles target glioblastoma. Chem. Sci..

[20] Bondeson D.P., Mullin-Bernstein Z., Oliver S., Skipper T.A., Atack T.C., Bick N., Ching M., Guirguis A.A., Kwon J., Langan C., Millson D., Paolella B.R., Tran K., Wie S.J., Vazquez F., Tothova Z., Golub T.R., Sellers W.R., Ianari A. (2022). Systematic profiling of conditional degron tag technologies for target validation studies. Nat. Commun..

[21] Gisterå A., Ketelhuth D.F.J., Malin S.G., Hansson G.K. (2022). Animal models of atherosclerosis–supportive notes and tricks of the trade. Circ. Res..

[22] Sanchez-Gaytan B.L., Fay F., Lobatto M.E., Tang J., Ouimet M., Kim Y., van der Staay S.E., van Rijs S.M., Priem B., Zhang L., Fisher E.A., Moore K.J., Langer R., Fayad Z.A., Mulder W.J. (2015). HDL-Mimetic PLGA nanoparticle to target atherosclerosis plaque macrophages. Bioconjug. Chem..

[23] Sei Y.J., Ahn J., Kim T., Shin E., Santiago-Lopez A.J., Jang S.S., Jeon N.L., Jang Y.C., Kim Y. (2018). Detecting the functional complexities between high-density lipoprotein mimetics. Biomaterials.

[24] Marrache S., Dhar S. (2013). Biodegradable synthetic high-density lipoprotein nanoparticles for atherosclerosis. Proc. Natl. Acad. Sci..

[25] Huynh E., Zheng G. (2014). Organic biophotonic nanoparticles: porphysomes and beyond. IEEE J. Sel. Top. Quant. Electron..

[26] VanderLaan P.A., Reardon C.A., Getz G.S. (2004). Site specificity of atherosclerosis. Arterioscler. Thromb. Vasc. Biol..

[27] Sanchez-Gaytan B.L., Fay F., Lobatto M.E., Tang J., Ouimet M., Kim Y., van der Staay S.E.M., van Rijs S.M., Priem B., Zhang L., Fisher E.A., Moore K.J., Langer R., Fayad Z.A., Mulder W.J.M. (2015). HDL-mimetic PLGA nanoparticle to target atherosclerosis plaque macrophages. Bioconjug. Chem..

[28] Ley K., Miller Y.I., Hedrick C.C. (2011). Monocyte and macrophage dynamics during atherogenesis. Arterioscler. Thromb. Vasc. Biol..

[29] Grebe A., Hoss F., Latz E. (2018). NLRP3 inflammasome and the IL-1 pathway in atherosclerosis. Circ. Res..

[30] Ridker P.M., Everett B.M., Thuren T., MacFadyen J.G., Chang W.H., Ballantyne C., Fonseca F., Nicolau J., Koenig W., Anker S.D., Kastelein J.J.P., Cornel J.H., Pais P., Pella D., Genest J., Cifkova R., Lorenzatti A., Forster T., Kobalava Z., Vida-Simiti L., Flather M., Shimokawa H., Ogawa H., Dellborg M., Rossi P.R.F., Troquay R.P.T., Libby P., Glynn R.J. (2017). Antiinflammatory therapy with canakinumab for atherosclerotic disease. N. Engl. J. Med..

[31] Libby P. (2017). Interleukin-1 beta as a target for atherosclerosis therapy: biological basis of CANTOS and beyond. J. Am. Coll. Cardiol..

[32] Baldrighi M., Mallat Z., Li X. (2017). NLRP3 inflammasome pathways in atherosclerosis. Atherosclerosis.

[33] Swanson K.V., Deng M., Ting J.P.Y. (2019). The NLRP3 inflammasome: molecular activation and regulation to therapeutics. Nat. Rev. Immunol..

[34] Döring Y., van der Vorst E.P.C., Yan Y., Neideck C., Blanchet X., Jansen Y., Kemmerich M., Bayasgalan S., Peters L.J.F., Hristov M., Bidzhekov K., Yin C., Zhang X., Leberzammer J., Li Y., Park I., Kral M., Nitz K., Parma L., Gencer S., Habenicht A.J.R., Faussner A., Teupser D., Monaco C., Holdt L., Megens R.T.A., Atzler D., Santovito D., von Hundelshausen P., Weber C. (2024). Identification of a non-canonical chemokine-receptor pathway suppressing regulatory T cells to drive atherosclerosis. Nature Cardiovascular Research.

[35] Weber C., Meiler S., Döring Y., Koch M., Drechsler M., Megens R.T., Rowinska Z., Bidzhekov K., Fecher C., Ribechini E., van Zandvoort M.A., Binder C.J., Jelinek I., Hristov M., Boon L., Jung S., Korn T., Lutz M.B., Förster I., Zenke M., Hieronymus T., Junt T., Zernecke A. (2011). CCL17-expressing dendritic cells drive atherosclerosis by restraining regulatory T cell homeostasis in mice. J. Clin. Investig..

[36] Yan Y., Thakur M., van der Vorst E.P.C., Weber C., Döring Y. (2021). Targeting the chemokine network in atherosclerosis. Atherosclerosis.

[37] Rousselle A., Qadri F., Leukel L., Yilmaz R., Fontaine J.F., Sihn G., Bader M., Ahluwalia A., Duchene J. (2013). CXCL5 limits macrophage foam cell formation in atherosclerosis. J. Clin. Investig..

[38] Gordon T., Castelli W.P., Hjortland M.C., Kannel W.B., Dawber T.R. (1977). High density lipoprotein as a protective factor against coronary heart disease. The framingham study. Am. J. Med..

[39] Rohatgi A., Westerterp M., von Eckardstein A., Remaley A., Rye K.-A. (2021). HDL in the 21st century: a multifunctional roadmap for future HDL research. Circulation.

[40] Chen R., Sandeman L., Nankivell V., Tan J.T.M., Rashidi M., Psaltis P.J., Zheng G., Bursill C., McLaughlin R.A., Li J. (2024). Detection of atherosclerotic plaques with HDL-Like porphyrin nanoparticles using an intravascular dual-modality optical coherence tomography and fluorescence system. Sci. Rep..

