# Supplementary material for: Theranostic porphyrin nanoparticles identify atherosclerosis via multimodal imaging and elicit atheroprotective effects
Source: Mater Today Bio. 2025 Aug 21;34:102202. doi: 10.1016/j.mtbio.2025.102202 (PMC12398886; doi:10.1016/j.mtbio.2025.102202)
Supplement: Multimedia component 1 [file mmc1.docx]

# ­­Theranostic porphyrin nanoparticles identify atherosclerosis via multimodal imaging and elicit atheroprotective effects

Victoria A. Nankivell, BSc PhD^a,b,c^, Lauren Sandeman, BSc^a,c^, Liam Stretton, BSc^a,c^, Achini K. Vidanapathirana, MBBS PhD^a,b,c^, Maneesha A. Rajora, MASc^d,e^, Juan Chen, BSc PhD^d,e^, William Tieu, BSc PhD^a^, Hanyi Weng^d,e^, BSc, Maaike Kockx^f^, BSc PhD, Leonard Kritharides^f,g^, MBBS PhD, Peter J. Psaltis, MBBS PhD^a,b,c^, Joanne T. M. Tan, BSc PhD^a,c^, Yung-Chih Chen, BSc PhD^h^, Karlheinz Peter, MBBS PhD^h^, Gang Zheng, BSc PhD^d,e^, Christina A. Bursill, BSc PhD^a,b,c*^

^a^ Vascular Research Centre, Lifelong Health Theme, South Australian Health and Medical Research Institute, Adelaide, South Australia, Australia 5000.

^b^ Australian Research Council (ARC) Centre of Excellence for Nanoscale BioPhotonics (CNBP)

^c^ Faculty of Health and Medical Science, Adelaide Medical School, University of Adelaide, Adelaide, South Australia, Australia 5000.

^d^ Princess Margaret Cancer Centre, University Health Network, Toronto, Ontario, M5G 1L7 Canada

^e^ Department of Medical Biophysics, University of Toronto, Ontario M5G 1L7, Canada.

^f^ ANZAC Research institute, Concord Repatriation General Hospital, Sydney Local Health district and University of Sydney

^g^Department of Cardiology, Concord Repatriation General Hospital, Sydney Local Health District and University of Sydney

^h^ Atherothrombosis and Vascular Biology, Baker Heart and Diabetes Institute, Melbourne, Victoria, Australia

**Running title:**  Theranostic nanoparticles for atherosclerosis

**Address for Correspondence**: A/Prof Christina Bursill

Vascular Research Centre, Lifelong Health Theme,

South Australian Health and Medical Research Institute.

Adelaide Medical School, Faculty of Health and Medical Science, University of Adelaide.

North Terrace, Adelaide, 5000, South Australia, Australia

**Email:** Christina.Bursill@sahmri.com

**Tel.:** +61 881 284 788

**Category:** Original Research

**Word Count:** 7767

**Contents - SUPPLEMENTAL MATERIALS**

Supplemental Figure S1. Characterization of R4F peptide and porphyrin lipid components of Porphyrin-lipid NPs.

Supplemental Figure S2: Detection of Por-NPs internalized by iBMDM macrophages using flow cytometry.

Supplemental Figure S3: Overexpression of hSR-BI increases Por-NP uptake in CHO cells

Supplemental Figure S4: Effect of Por-NPs on pro- inflammatory mediators in iBMDMs

Supplemental Figure S5: Confirmation of siRNA knockdown of SR-BI and stimulation of cholesterol efflux by methyl-β-cyclodextrin in iBMDM macrophages.

Supplemental Figure S6: Por-NPs suppress components of the NLRP3 inflammasome.

Supplemental Figure S7: Biodistribution of Por-NPs in chow and HCD fed *Apoe^-/-^* mouse organs detected *ex vivo* using fluorescence imaging.

Supplemental Figure S8: Effect of R4F peptide on fluorescence uptake and detection *in vitro* and *in vivo*

Supplemental Figure S9: Schematics representing experimental protocols of early-stage stable plaque, mid-late-stage plaque and tandem stenosis unstable plaque mouse models.

Supplemental Figure S10: Effect of Por-NP on plaque composition.

Supplemental Figure S11: Effect of Por-NPs on pro- inflammatory mediators in aortic arches.

Supplemental Table S1. Compositions of porphyrin lipid nanoparticles.

Supplemental Table S2: Summary of mean size and polydispersity index.

Supplemental Table S3. Murine primer sequences used for quantitative PCR in iBMDMs and aortic arches.

Supplemental Table S4. Flow cytometry antibodies.

Supplemental Table S5. Plasma lipid measures in early-stage atherosclerosis model.

Supplemental Table S6. Plasma lipid measures in tandem stenosis unstable plaque model.

Supplemental Table S7. Lipid film compositions for nanoparticle formulations

# SUPPLEMENTAL MATERIALS


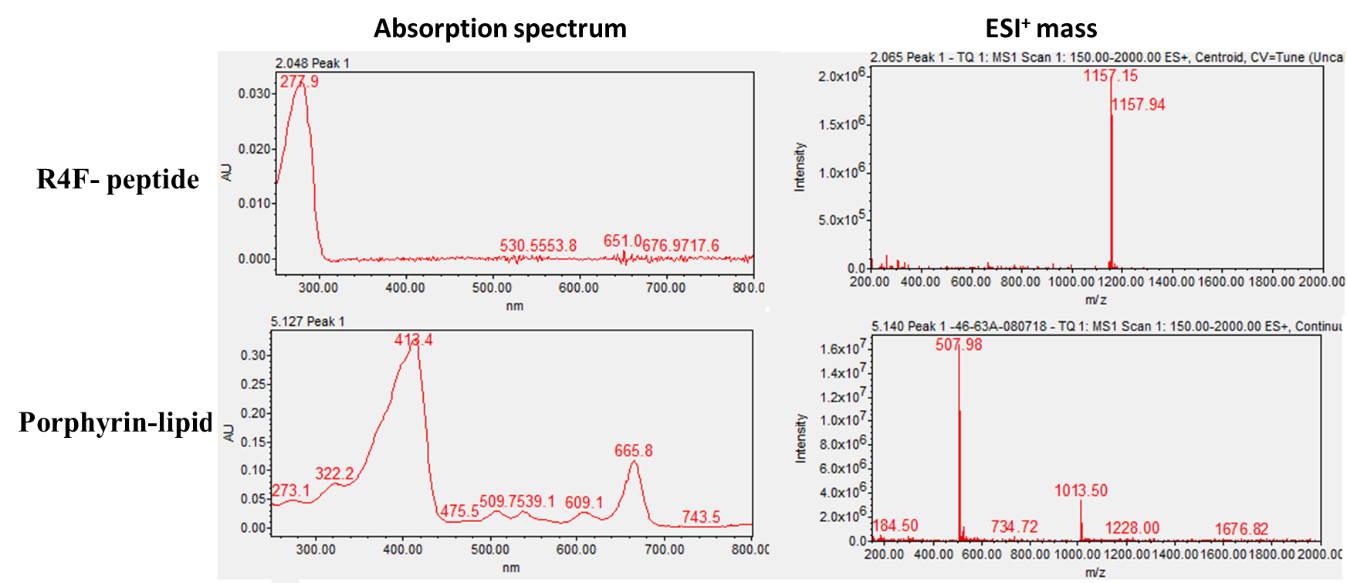


**Figure S1**. **Characterization of R4F peptide and porphyrin lipid components of Porphyrin-lipid NPs. (A)** Characterisation by absorption spectra (left column) and mass spectrometry (right column). R4F peptide: molecular formula C114H156N24O28, molecular weight 2310.60; ESI+ mass observed: [M]2+ = 1157.15. Porphyrin lipid: molecular formula C57H82N5O9P, molecular weight 1012.26; ESI+ mass observed: [M]2+ = 507.98, [M]+ = 1013.50. ESI, Electrospray ionization. (**B**) ^1^H nuclear magnetic resonance (NMR) spectra for the porphyrin-lipid.


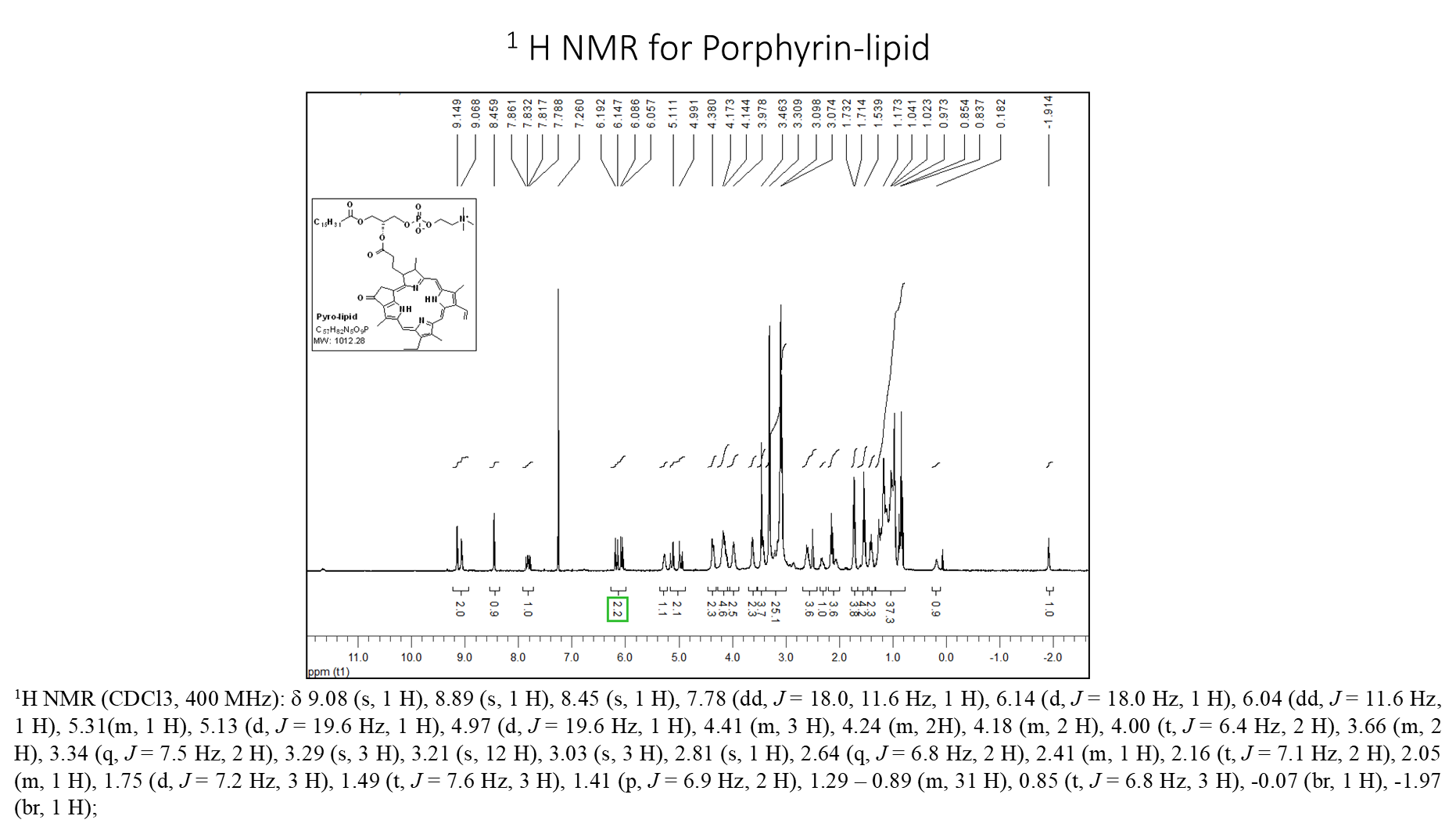


**A**

**B**

**^1^H NMR for porphyrin lipid**

**Supplemental Figure S1**

**Supplemental Figure S2**

**
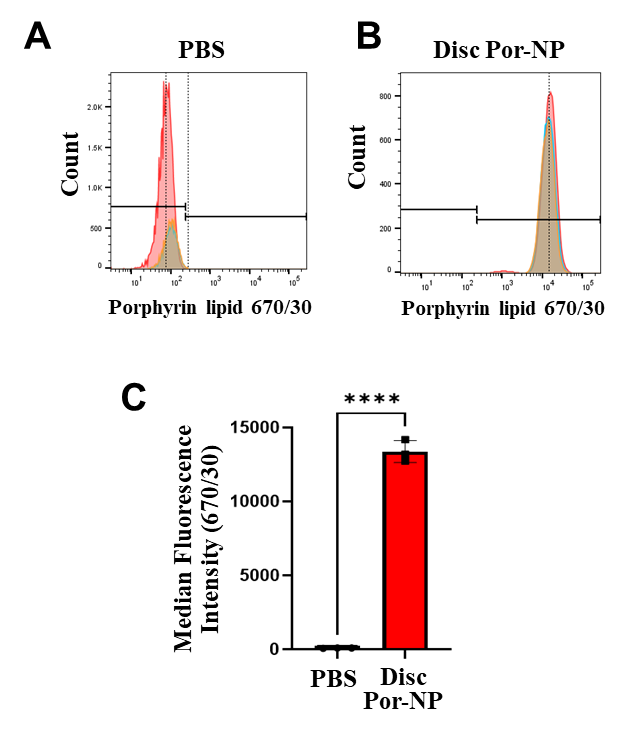
**

**Supplemental Figure S2: Detection of Por-NPs internalized by iBMDM macrophages using flow cytometry.**

**(A-B)** iBMDMs were incubated with PBS or discoidal Por-NPs (10 µg/mL) for 24h then analyzed by flow cytometry with the red laser (excitation λ: 640 nm; emission λ: 670/30 nm) to detect porphyrin-lipid fluorescence. **(C)** Median fluorescence intensity (MFI) was quantified from flow cytometric analysis of the porphyrin-lipid fluorescence within iBMDMs. Data expressed as Mean ± SD (n=3 biological replicates), ****P*<0.0001 vs PBS control by two-tailed unpaired t-test.

**Supplemental Figure S3**

**
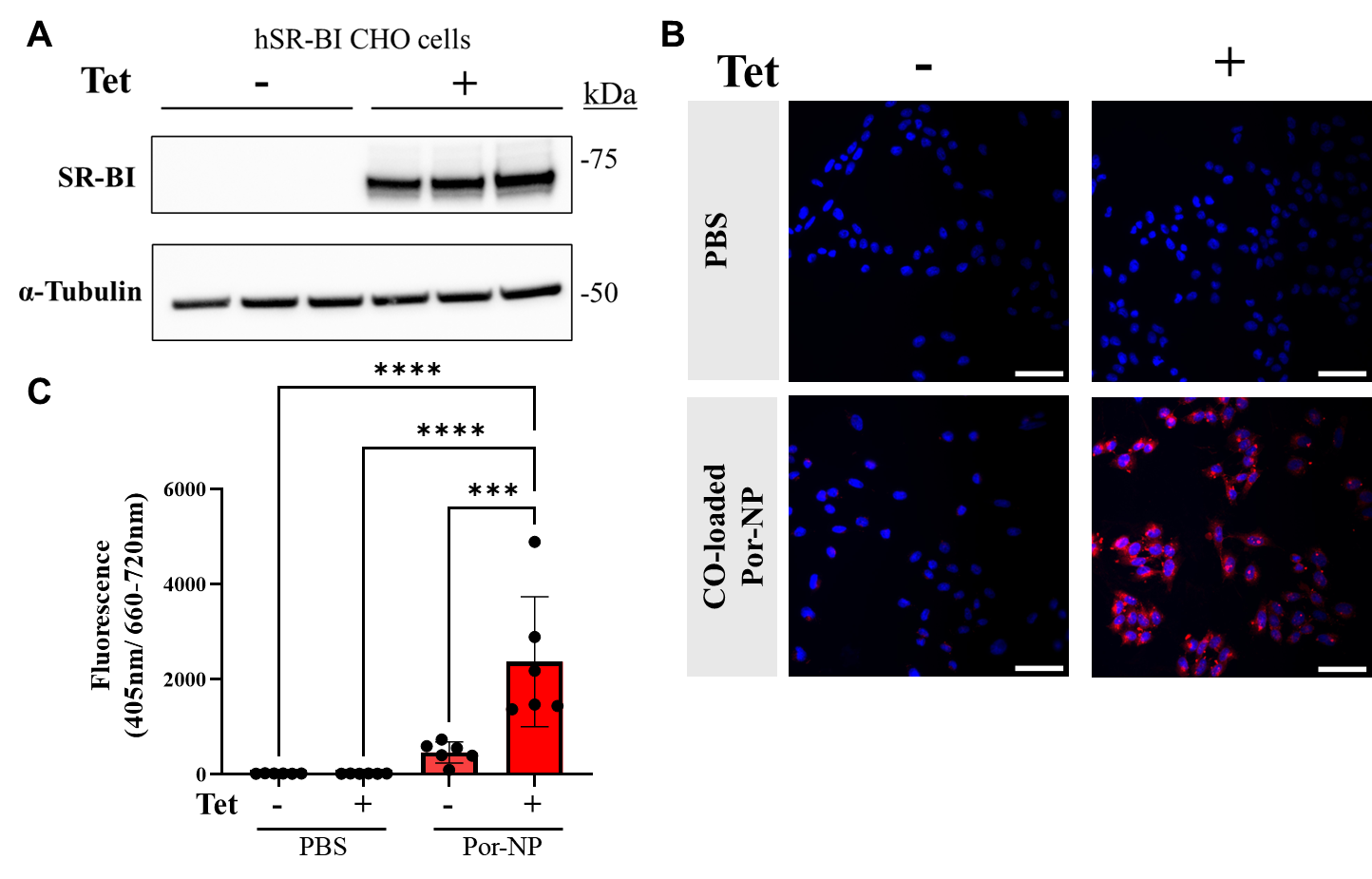
**

Supplemental Figure S3: Overexpression of hSR-BI increases Por-NP uptake in CHO cells

Chinese Hamster Ovary (CHO) cells with tetracycline (Tet) inducible expression of human SR-BI (hSR-BI) were incubated with (+) and without (-) tetracycline in cell culture media for 18h then incubated with PBS or Por-NPs for 3h. **(A)** Western blotting to confirm overexpression of hSR-BI in CHO cells treated with tetracycline. **(B)** Representative fluorescence microscopy images of CHO cells with Por-NP uptake in red and nuclei in blue (DAPI). Scale bar: 100μm. (C) Quantification of Por-NP fluorescence in treated CHO cell lysates (excitation wavelength: 405nm, emission wavelength 660-720nm). Data expressed as Mean ± SD (n=6). ****P*<0.001 and *****P*<0.0001 by one-way ANOVA with post-hoc Tukey’s multiple comparisons.

**
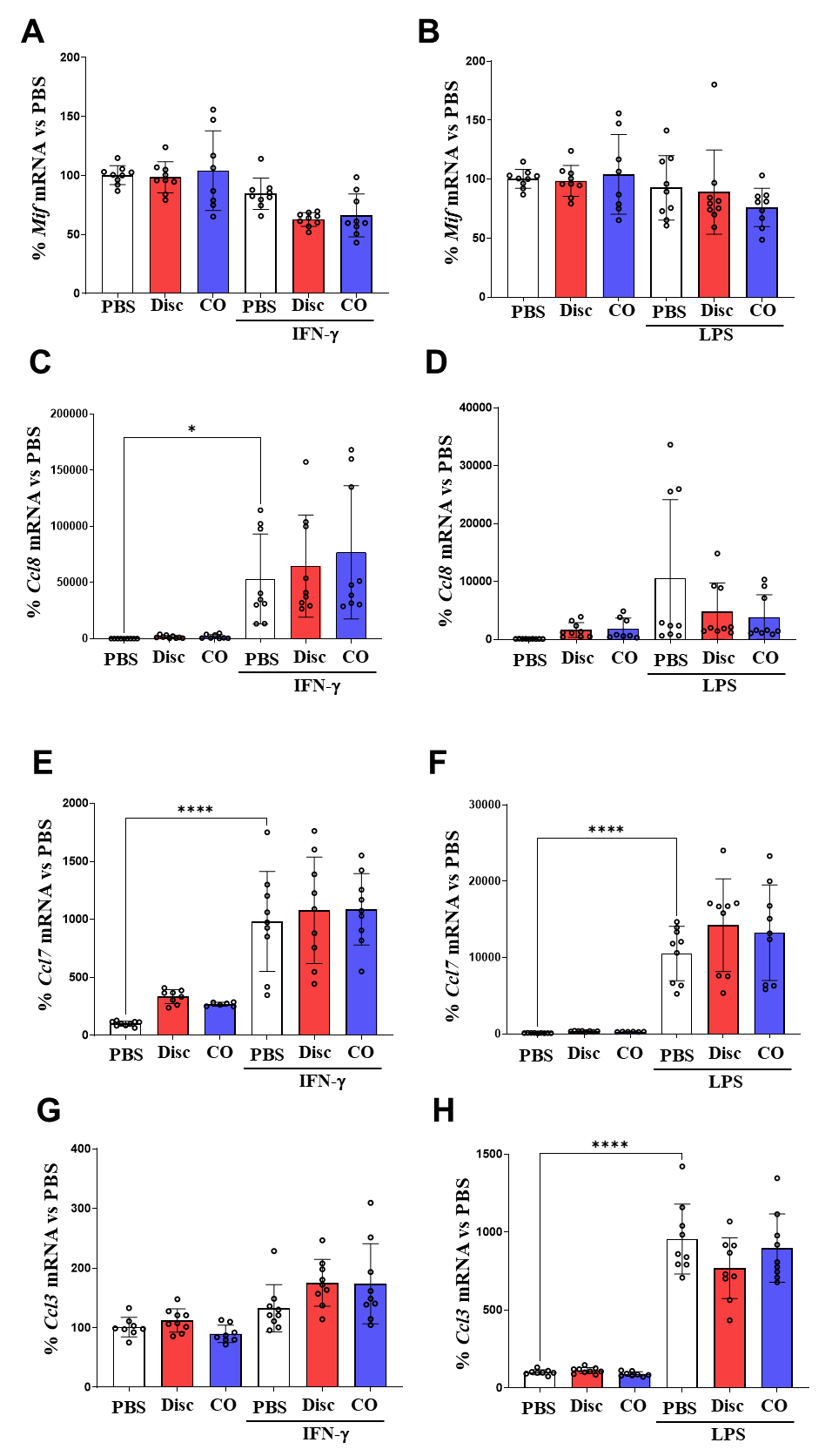
Supplemental Figure S4**

Supplemental Figure S4: Effect of Por-NPs on pro- inflammatory mediators in iBMDMs

RT-qPCR measurements of **(A-B)** *Mif,* **(C-D)** *Ccl8,* **(E-F)** *Ccl7* and **(G-H)** *Ccl3* mRNA levels following incubation with discoidal (Disc) or CO-loaded (CO) Por-NPs and stimulation with IFN-γ (10 ng/mL) or LPS (10 ng/mL) (n=8-9 biological replicates). Data expressed as Mean ± SD. ***P*<0.01, ****P*<0.001, *****P*<0.0001 by one-way ANOVA with post-hoc Tukey’s multiple comparisons.

**
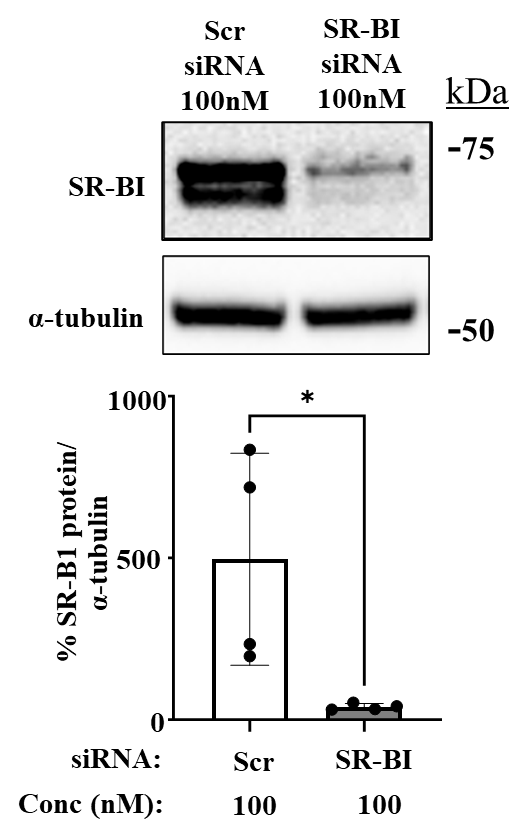
**
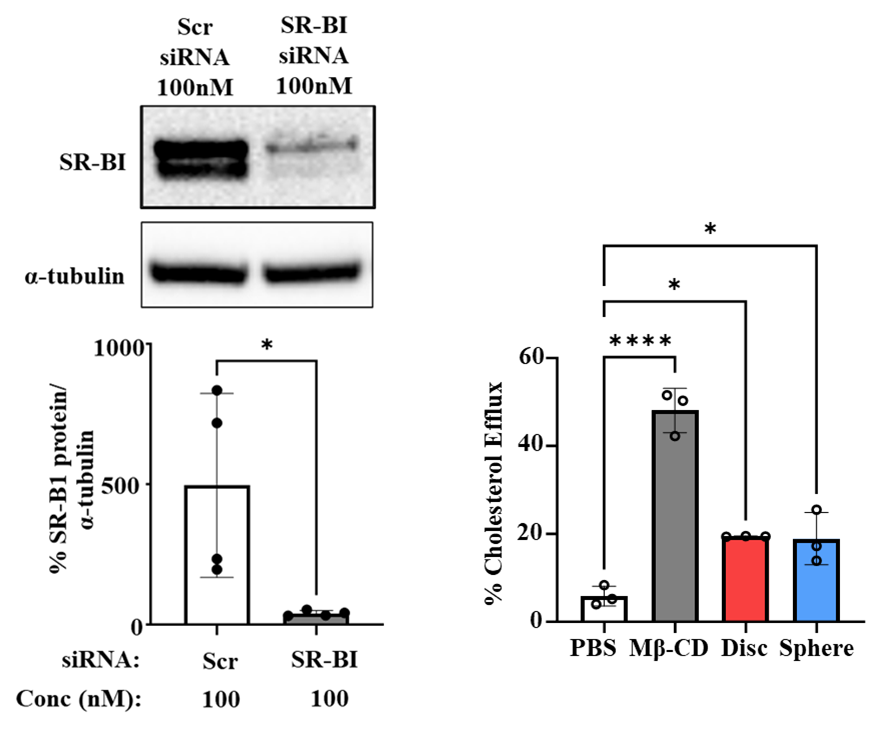
**Supplemental Figure S5**

**A**

**B**

**Supplemental Figure S5: Confirmation of siRNA knockdown of SR-BI and stimulation of cholesterol efflux by methyl-β-cyclodextrin in iBMDM macrophages.**

**(A)** iBMDMs were transfected with Scrambled (Scr) or SR-BI siRNA (100 nM). After a 48h incubation, iBMDMs were harvested for Western blot analyses of SR-BI protein on whole cell lysates (n=4 biological replicates). Western blot image (top panel) showing SR-BI (~70kDa observed) and α-tubulin (~55kDa observed) as the loading control. **P*<0.05 by unpaired t-test. **(B)** Cholesterol efflux was measured in iBMDMs following treatment with PBS, methyl-β-cyclodextrin (MβCD, 4% v/v), discoidal (Disc) or CO-loaded (Sphere) Por-NPs (25 μg/mL). n=3 biological replicates. All data expressed as Mean ± SD. **P*<0.05, *****P*<0.0001 vs PBS by one-way ANOVA with post-hoc Dunnett’s multiple comparisons.

**Supplemental Figure S6**

**
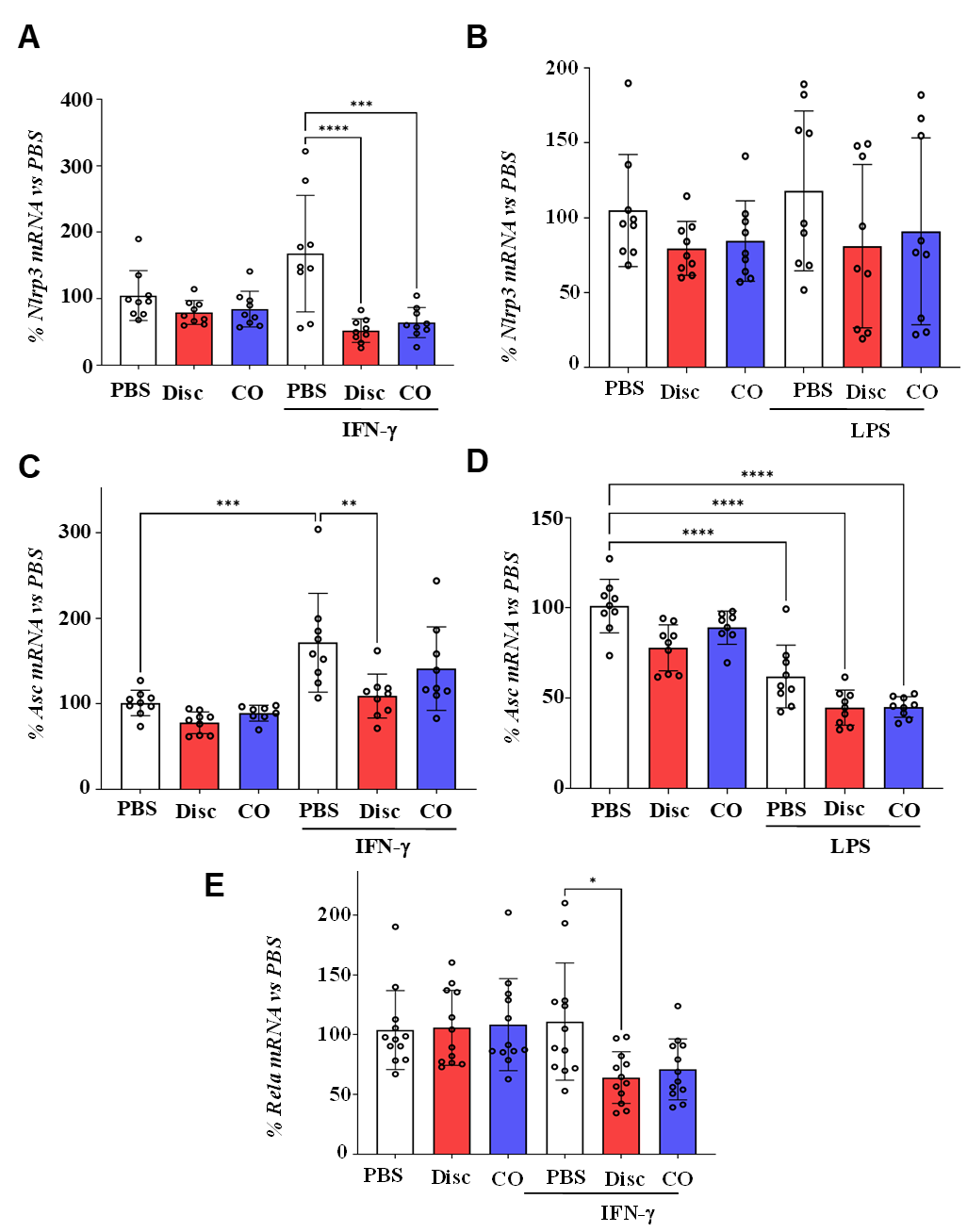
**

Supplemental Figure S6: Por-NPs suppress components of the NLRP3 inflammasome.

RT-qPCR measurements of **(A-B)** *Nlrp3* and **(C-D)** *Asc* mRNA levels following incubation with discoidal (Disc) or CO-loaded (CO) Por-NPs and stimulation with IFN-γ (10 ng/mL) or LPS (10 ng/mL) (n=9-12 biological replicates). Data expressed as Mean ± SD. ***P*<0.01, ****P*<0.001, *****P*<0.0001 by one-way ANOVA with post-hoc Tukey’s multiple comparisons.

**Supplemental Figure S7**

**
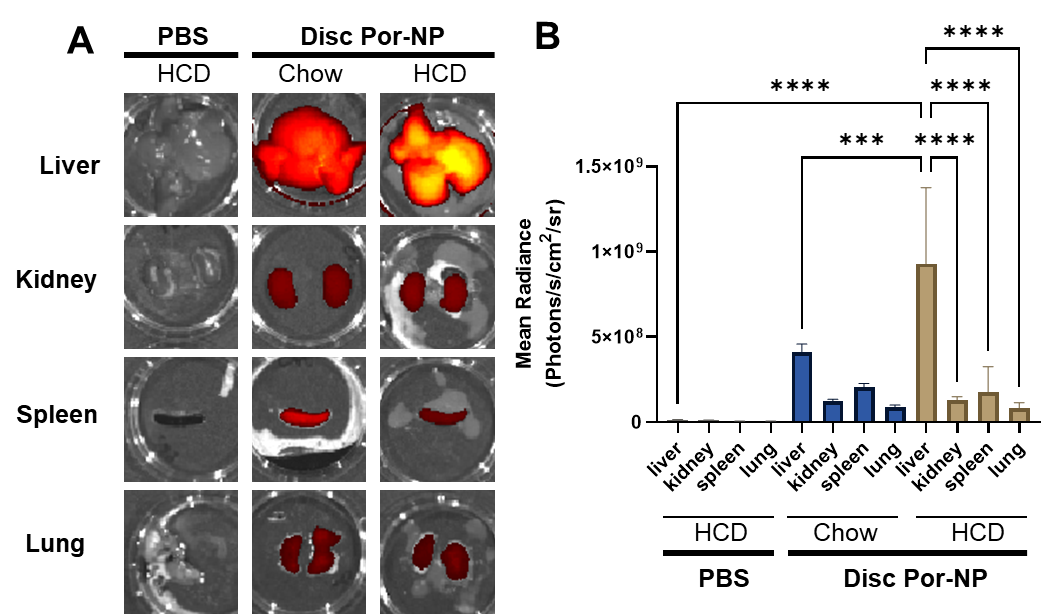
**

**Supplemental Figure S7: Biodistribution of Por-NPs in chow and HCD fed *Apoe^-/-^* mouse organs detected *ex vivo* using fluorescence imaging.**

**(A)** Representative images of excised liver, kidneys, spleen and lungs from *Apoe^-/-^* mice fed either a chow or HCD collected 24h following intraperitoneal injection of PBS or discoidal Por-NPs. **(B)** Fluorescence quantified from IVIS images of excised organs expressed as mean radiance (Photons/s/cm^2^/sr). Fluorescence images of Por-NP chow and HCD organs were captured side-by-side in a single image with the IVIS camera. n=1-4 animals/ group (PBS/Chow: n=1, HCD: n=4, Note: Chow n=4 technical replicates). Data expressed as Mean ± SD. ****P*<0.001, *****P*<0.0001 by one-way ANOVA with post-hoc Tukey’s multiple comparisons. IVIS: *In vivo* imaging system; s: second, sr: steradian.

**
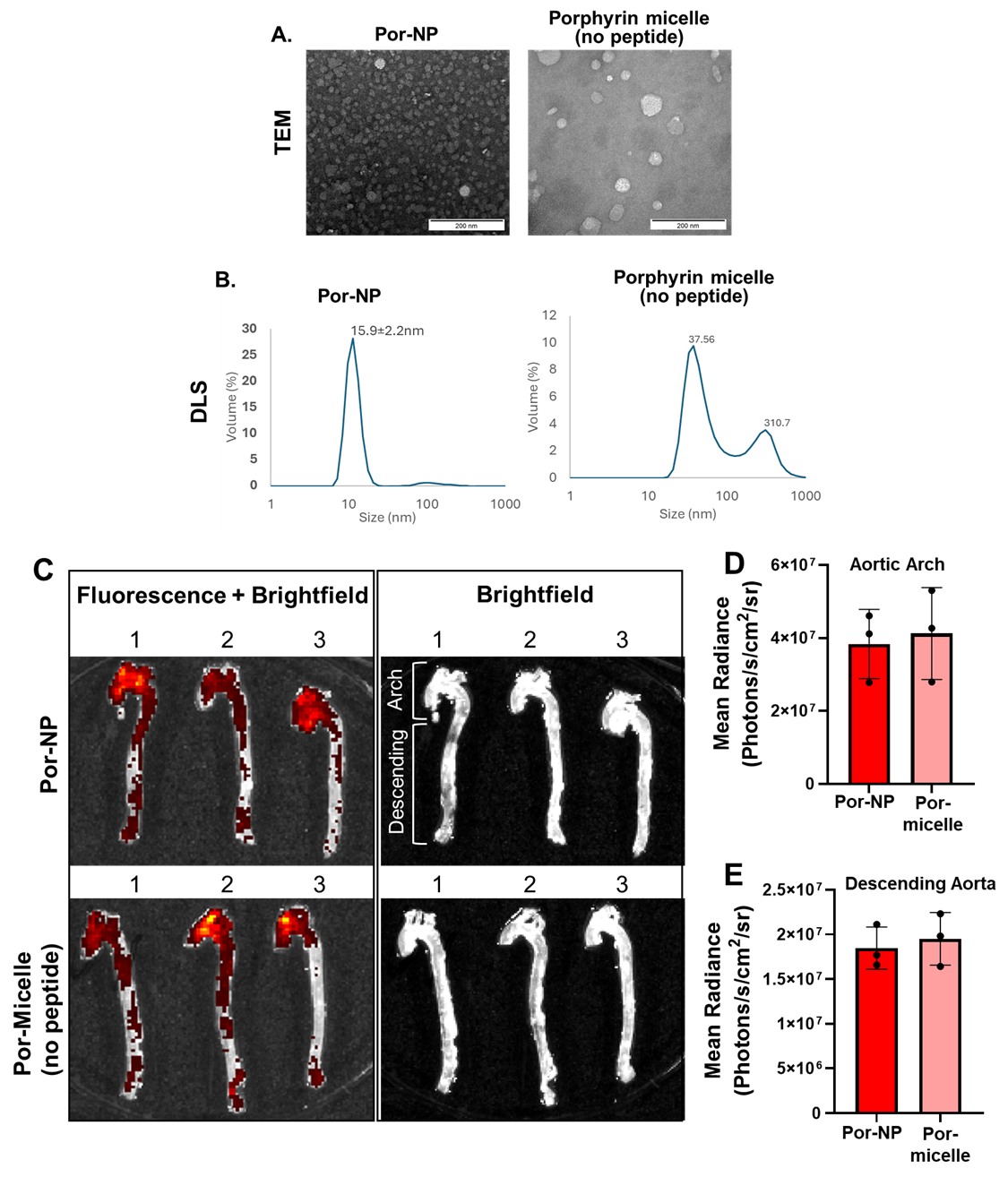
**

Supplemental Figure S8

**Supplemental Figure S8: Plaque uptake of Por-NPs versus porphyrin micelles (without R4F peptide) *in vivo.*** (**A**) Transmission electron microscope (TEM) images and (**B**) dynamic light scattering (DLS) of Por-NP and Por-micelles. (**C**) IVIS fluorescence images of aortas from *Apoe^-/-^* mice fed HCD for 9 weeks collected 24h following intraperitoneal injection of 5mg/kg Por-NPs or Por-micelles (no peptide). Fluorescence quantified from IVIS images for (**D**) aortic arch and (**E**) descending aorta, expressed as mean radiance (Photons/s/cm^2^/sr). Fluorescence images of aortas were captured side-by-side in a single image with the IVIS camera. Data expressed as Mean ± SD (n=3). IVIS: In vivo imaging system; s: second, sr: steradian.


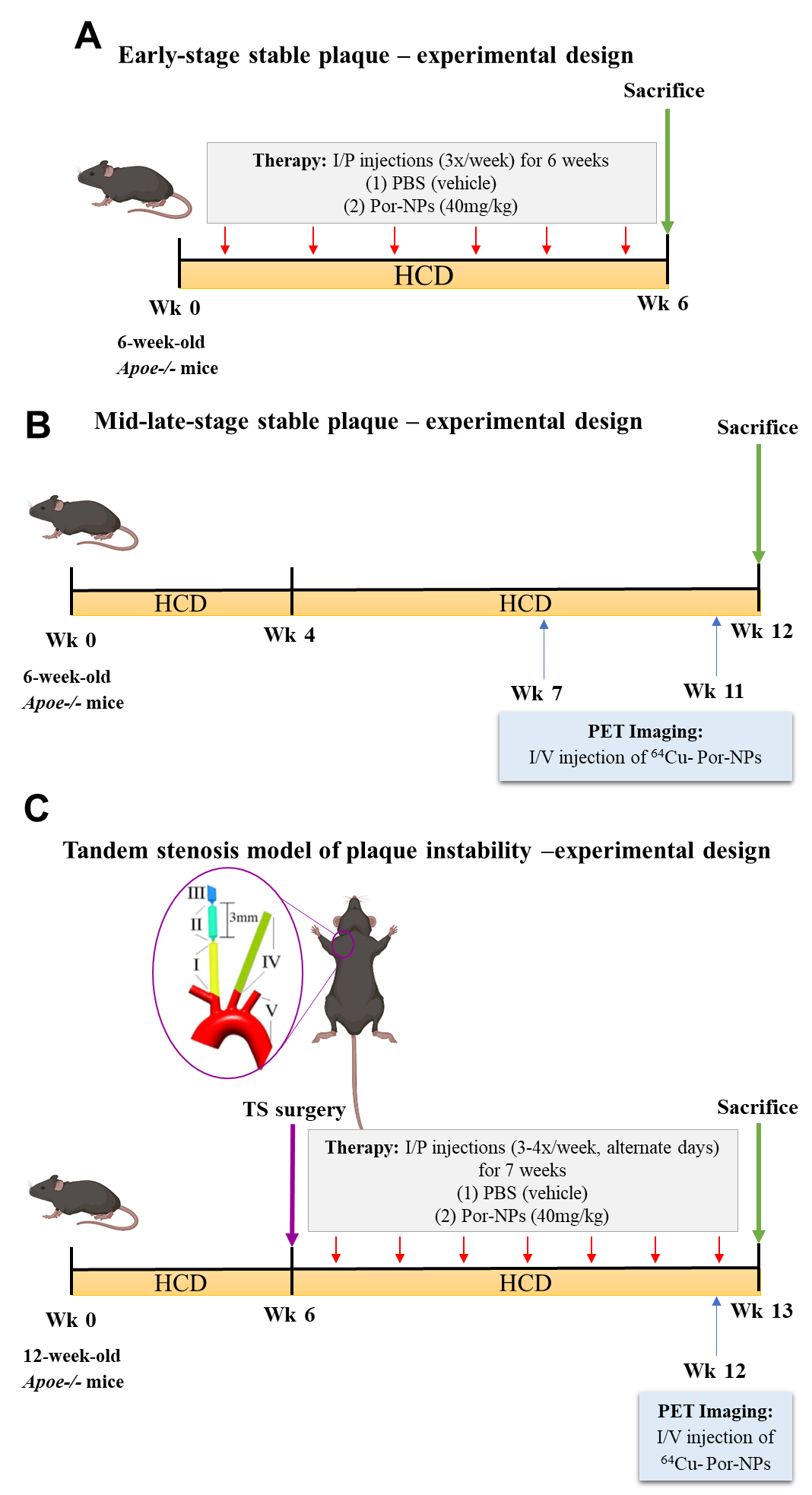


Supplemental Figure S9

**Supplemental Figure S9: Schematics representing experimental protocols of early-stage stable plaque, mid-late-stage plaque and tandem stenosis unstable plaque mouse models.**

Animals received 3 times per week or 3-4 times per week (alternate days) intraperitoneal (I/P) injections of discoidal Por-NPs for therapeutic intervention in **(A)** an early-stage atherosclerosis model, (**B**) mid-late-stage atherosclerosis model that included intravenous (I/V) infusions of ^64^Cu-Por-NPs for serial PET imaging in week 7 and 11, and **(C)** the tandem stenosis model of plaque instability that included I/V infusions of ^64^Cu-Por-NPs for PET imaging in week 12.


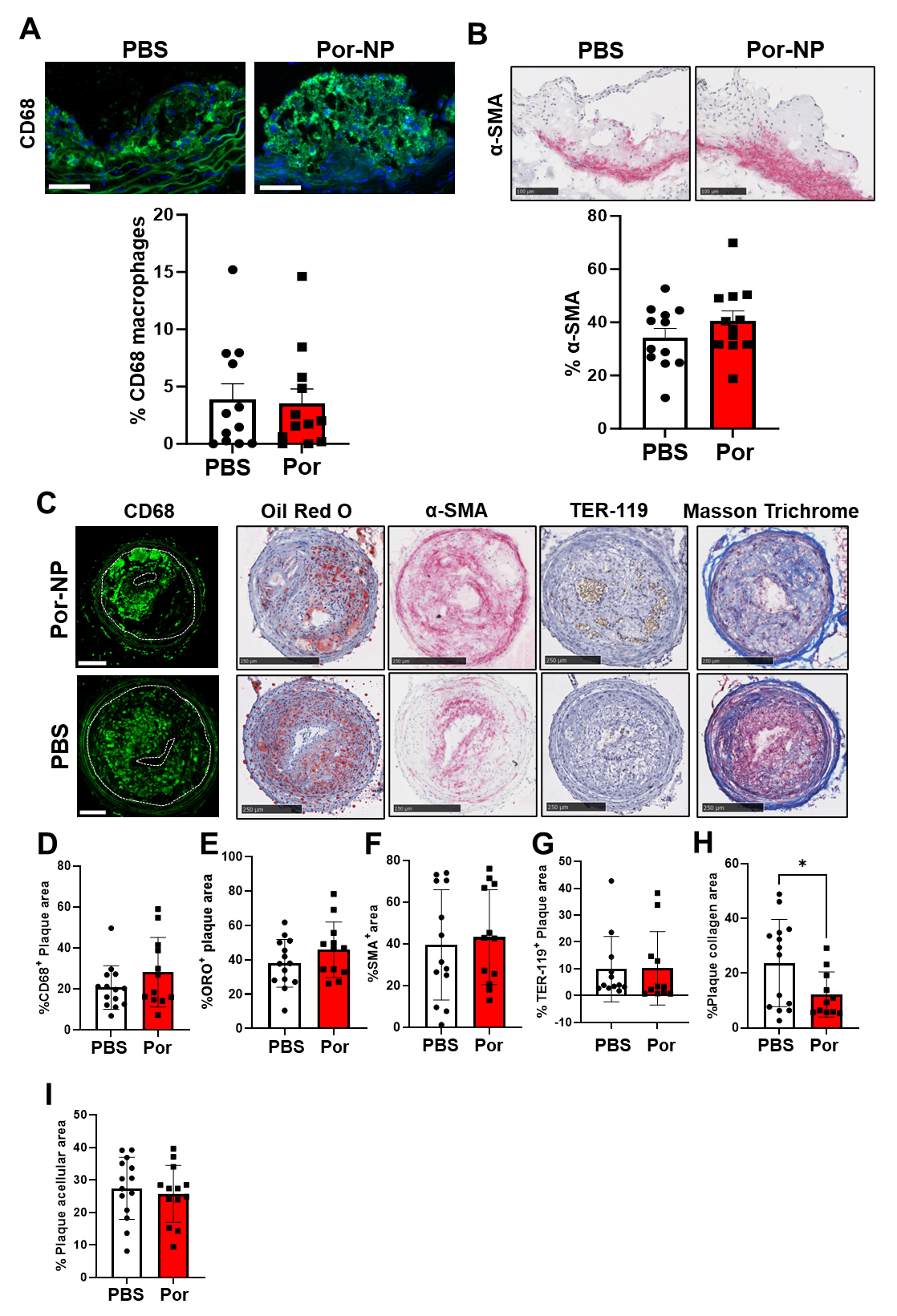


Supplemental Figure S10

Supplemental Figure S10: Effect of Por-NP on plaque composition.

Six-week-old *Apoe*^-/-^ mice were fed HCD for 6 weeks to develop early-stage plaque in the aortic sinus and received PBS or Por-NP infusions on alternate days intraperitoneally. Analysis and representative microscopy images of aortic sinus sections assessed for: (A) CD68^+^ macrophages (Scale bar: 50 µm) and (B) α-SMA^+^ smooth muscle cells (Scale bar: 100 µm), calculated as a % of total plaque area. (C) After 6 weeks of HCD, *Apoe*^-/-^ mice received tandem stenosis carotid artery ligation surgery. The mice were then infused with either PBS or Por-NPs on alternate days intraperitoneally for a further 7 weeks. Representative images of fluorescence and light microscopy of carotid artery sections to detect: (D) CD68^+^ macrophages, (E) Oil Red O lipid, (F) α-SMA^+^ smooth muscle cells and (G) TER-119^+^ erythrocytes (H) Collagen (Masson’s trichrome). (I) % Plaque acellular measured from unstained regions of H&E sections. (J) Area of vessel measured from Internal Elastic Lamina (IEL area) measured from H&E (Scale bar:100μm) and average IEL area calculated from three locations spanning Segment I of the carotid. Scale bar: CD68 = 200 µm; Oil Red O, TER-119, α-SMA, MT = 250 µm. Data expressed as mean ± SD (n=12-14 animals/group). **P*<0.05 vs PBS control by two-tailed unpaired t-test. Por: Por-NP.

**Supplemental Figure S11
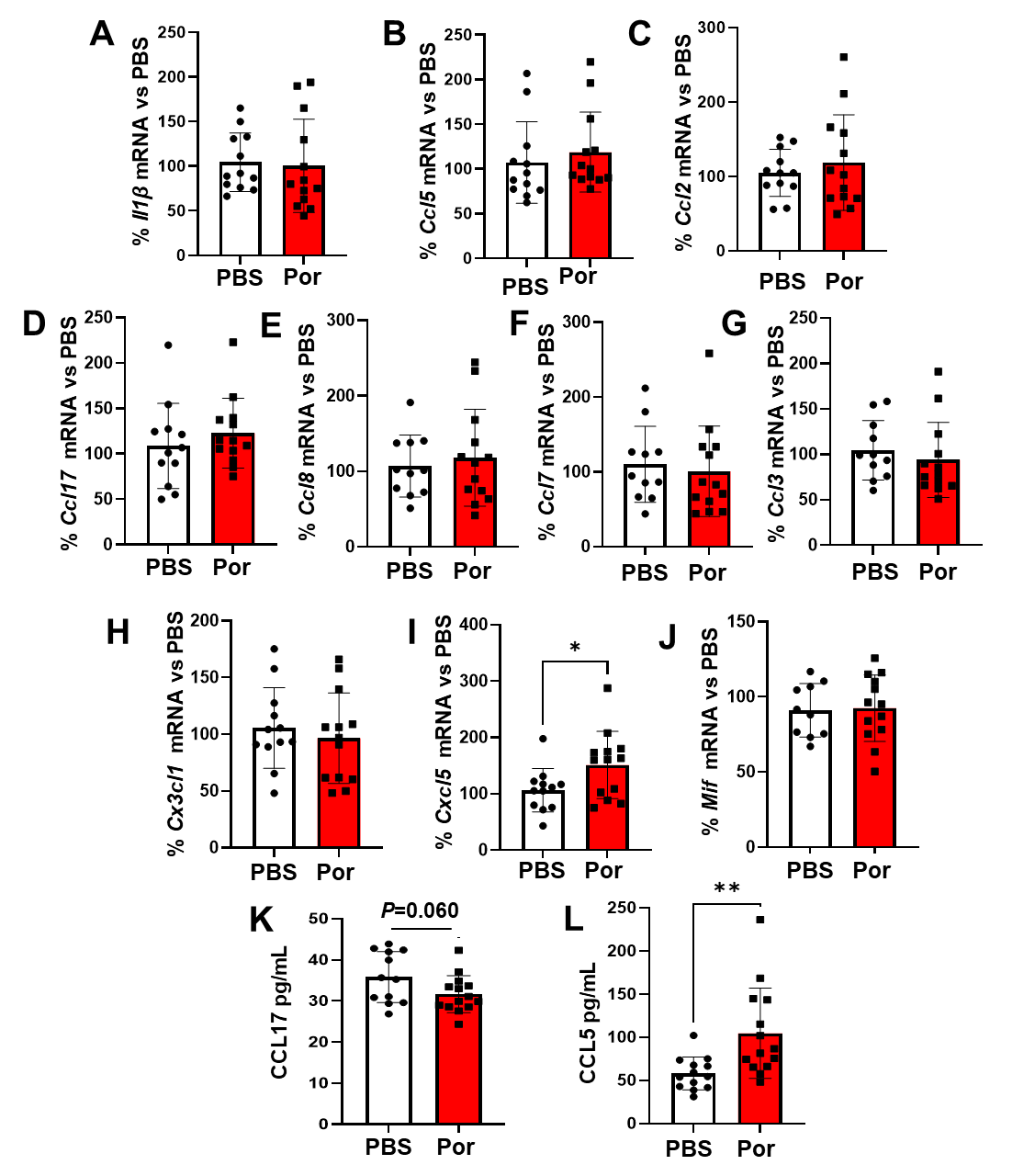
**

Supplemental Figure S11: Effect of Por-NPs on pro- inflammatory mediators in aortic arches

RT-qPCR measurements of **(A)** *Il1b*, **(B)** *Ccl5*, **(C)** *Ccl2*, **(D)** *Ccl17*, **(E)** *Ccl8*, **(F)** *Ccl7*, **(G)** *Ccl3*, **(H)** *Cx3cl1*, **(I)** *Cxcl5* and **(J)** *Mif,* from mRNA isolated from the aortic arches, and plasma **(K)** CCL17 and **(L)** CCL5 by ELISA from *Apoe*^-/-^ mice fed HCD for 6 weeks prior to tandem stenosis surgery and intraperitoneal injection of PBS or Por-NPs on alternate days for 7 weeks. Data expressed as Mean ± SD. **P*<0.05 by Student T-test.

**Supplemental Table S1**

**
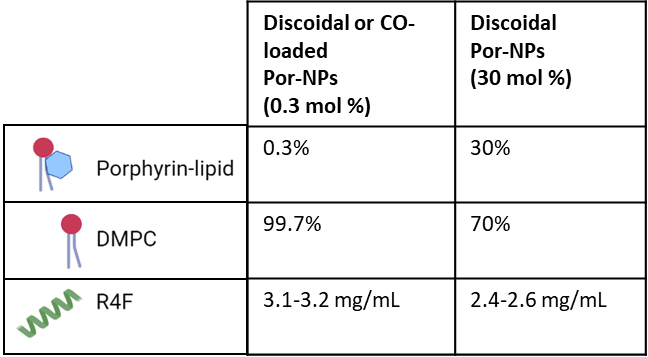
**

**Supplemental Table S1**. **Compositions of porphyrin lipid nanoparticles**

Porphyrin-lipid nanoparticle (Por-NP) compositions for cholesteryl oleate (CO) spherical-like Por-NPs and discoidal Por-NPs with variations in porphyrin-lipid, 1,2-Dimyristoyl-sn-glycero-3-phosphocholine (DMPC) and R4F peptide. DMPC phospholipid (% of total moles of lipid per particle) outlined for each nanoparticle formulation.

**Supplemental Table S2**

| **Formulation** | **Number of particles analyzed** | **Average size (nm) ± SD of combined histogram** | **Median (nm)** | **Calculated PDI from individual histograms averaged** |
| --- | --- | --- | --- | --- |
| **CO-loaded Por-NPs** | 720 | 21 ± 7 | 21 | 0.10±0.02 |
| **Discoidal Por-NPs** | 397 | 22 ± 6 | 22 | 0.06±0.02 |

**Supplemental Table S2: Summary of mean size and polydispersity index based on cumulative statistics derived from combined histograms across three batches for each formulation, visualized by transmission electron microscopy.**

**Supplemental Table S3**

| **Murine qPCR Primers** | **Forward (5'-3')** | **Reverse (5'-3')** |
| --- | --- | --- |
| ***Rplp0*** | CAACGGCAGCATTTATAACCC | CCCATTGATGATGGAGTGTGG |
| ***B2m*** | TTCTGGTGCTTGTCTCACTGA | CAGTATGTTCGGCTTCCCATTC |
| ***Rela (p65-Nfkb)*** | AGTATCCATAGCTTCCAGAACC | ACTGCATTCAAGTCATAGTCC |
| ***Nfkb1 (p50-Nfkb)*** | CCCTAAAGATTGTGCCAAGAG | GAAAGAGGTTATCCTGAAATCCC |
| ***Il1b*** | ACCTGTCCTGTGTAATGAAAGACG | TGGGTATTGCTTGGGATCC |
| ***Il18*** | GACTCTTGCGTCAACTTCAAGG | CAGGCTGTCTTTTGTCAACGA |
| ***Asc*** | CTTGTCAGGGGATGAACTCAAAA | GCCATACGACTCCAGATAGTAGC |
| ***Nlrp3*** | ATCAACAGGCGAGACCTCTG | GTCCTCCTGGCATACCATAGA |
| ***Scarb1 (Sr-b1)*** | CTGAGCACGTTCTACACGCA | GGCCTGAATGGCCTCCTTAT |
| ***Cx3cl1*** | CAGCAGTGACCGGATCATCTC | TGCTCTGAGGCTTAGCCGTAA |
| ***Cxcl5*** | TCCTCAGTCATAGCCGCAAC | ATGACTTCCACCGTAGGGCA |
| ***Mif*** | TACGACATGAACGCTGCCAA | AAGAACAGCGGTGCAGGTAA |
| ***Ccl2*** | GCTGGAGCATCCACGTGTT | ATCTTGCTGGTGAATGAGTAGCA |
| ***Ccl3*** | CCAAGTCTTCTCAGCGCCAT | GAATCTTCCGGCTGTAGGAGAAG |
| ***Ccl5*** | GCAAGTGCTCCAATCTTGCA | CTTCTCTGGGTTGGCACACA |
| ***Ccl7*** | GGGAAGCTGTTATCTTCAAGACAAA | CTCCTCGACCCACTTCTGATG |
| ***Ccl8*** | GCTACGAGAGAATCAACAATATCCAGT | CAGAGAGACATACCCTGCTTGGT |
| ***Ccl17*** | GGATGCCATCGTGTTTCTGA | TTCTTCACATGTTTGTCTTTG |

**Supplemental Table S3. Murine primer sequences used for quantitative PCR in iBMDMs and aortic arches.**

*Rplp0, Ribosomal protein lateral stalk subunit P0;* *Rela, p65 active subunit of nuclear factor kappa B; Nfkb1,* *nuclear factor kappa B 1/ p50 subunit; Ccl, Chemokine (C-C motif) ligand*; *Il1b, Interleukin-1 beta*; *Il18, Interleukin-18;* *Asc, Apoptosis-associated speck-like protein containing a CARD;* *Nlrp3, NOD-, LRR- and pyrin domain-containing protein 3; Scarb1, Scavenger receptor class B type 1; Mif, Macrophage migration inhibitory factor; Cxcl5, C-X-C motif chemokine ligand 5; Cx3cl1, C-X3-C motif chemokine ligand 1.*

| Antibody/ Stain | Catalog #, Manufacturer |
| --- | --- |
| Anti-Actin, α-Smooth Muscle – FITC, Mouse monoclonal | F3777, Sigma-Aldrich Inc., MO, USA |
| APC-Cy™7 Rat Anti-CD11b | 557657, BD Biosciences, NJ, USA |
| Brilliant Violet 510™ anti-mouse Ly-6G/Ly-6C (Gr-1) Antibody | 108438, Biolegend, CA, USA |
| Brilliant Violet 785™ anti-mouse F4/80 | 123141, Biolegend, CA, USA |
| BV421 Rat Anti-Mouse CD45 | 563890, BD Biosciences, NJ, USA |
| BV421 Rat Anti-Mouse CD86 | 564198, BD Biosciences, NJ, USA |
| FITC anti-mouse CD192 (CCR2) Antibody | 150607, Biolegend, CA, USA |
| PE Rat Anti-CD11b | 553311, BD Biosciences, NJ, USA |
| PE/Cyanine7 anti-mouse CD206 (MMR) | 141720, Biolegend, CA, USA |
| PE-Cy™7 Rat Anti-Mouse Ly-6G | 560601, BD Biosciences, NJ, USA |
| PerCp-Cy^TM^5.5 Rat Anti-mouse CD31 | 562861, BD Biosciences, NJ, USA |
| PerCP-Cy™5.5 Rat Anti-Mouse TER-119/Erythroid Cells | 560512, BD Biosciences, NJ, USA |

**Supplemental Table S4**

**Supplemental Table S4. Flow cytometry antibodies**

**Supplemental Table S5**

| **Plasma Lipids** | **PBS** | **Por-NP** | **P value** |
| --- | --- | --- | --- |
| **Total Cholesterol (mmol/L)** | 11.5 ± 2.7 | 11.3 ± 2.6 | 0.84 |
| **LDL Cholesterol (mmol/L)** | 7.9 ± 3.3 | 5.6 ± 1.9 | 0.12 |
| **HDL Cholesterol (mmol/L)** | 4.1 ± 2.2 | 5.6 ± 2.4 | 0.18 |
| **Triglycerides (mmol/L)** | 5.9 ± 2.2 | 4.9 ± 1.5 | 0.22 |

**Supplemental Table S5. Plasma lipid measures in early-stage atherosclerosis model.**

*Apoe*^-/-^ mice were infused 3-times per week from 6 weeks of age with PBS or Por-NPs via intraperitoneal injection for 6 weeks whilst on HCD. Plasma lipids were measured by enzymatic assay. n = 12 animals/group. Two-tailed unpaired t-test. Mean ± SD.

**Supplemental Table S6**

| **Plasma Lipids** | **PBS** | **Por-NP** | **P value** |
| --- | --- | --- | --- |
| **Total Cholesterol (mmol/L)** | 17.8 ± 3.5 | 17.3 ± 3.9 | 0.73 |
| **LDL Cholesterol (mmol/L)** | 13.8 ± 4.7 | 13.5 ± 4.4 | 0.84 |
| **HDL Cholesterol (mmol/L)** | 4.0 ± 0.7 | 3.5 ± 0.7 | 0.07 |
| **Triglycerides (mmol/L)** | 4.8 ± 1.0 | 3.7 ± 0.8** | 0.004 |

**Supplemental Table S6. Plasma lipid measures in tandem stenosis unstable plaque model.**

*Apoe*^-/-^ mice fed HCD from 12 weeks of age. After 6 weeks, mice received tandem stenosis surgery. After surgery, mice were administered PBS or discoidal Por-NPs (40mg/kg of R4F) via intraperitoneal injection on alternate days for seven weeks. Plasma lipids were measured by enzymatic assay. n = 13-14 animals/group. Mean ± SD. ***P*<0.01 PBS vs Por-NP by two-tailed unpaired t-test.

# SUPPLEMENTAL METHODS

**Porphyrin-lipid nanoparticle synthesis**Porphyrin-lipid nanoparticles (Por-NPs) consist of an outer shell of the phospholipid 1,2-Dimyristoyl-sn-glycero-3-phosphocholine (DMPC), porphyrin-lipid and R4F. R4F is an apoA-I alpha helical mimetic peptide (Ac-FAEKFKEAVKDYFAKFWD). The porphyrin lipid was formed by an acylation reaction of pyropheophorbide-a (porphyrin) and single chain phospholipid 1-palmitoyl-2-hydroxy-sn-glycero-3-phosphocholine (16:0 Lyso PC) as previously described [1, 2]. Porphyrin-lipid structure was confirmed by ^1^H-nuclear magnetic resonance (^1^H-NMR). Discoidal and cholesterol oleate (CO)-loaded [3] Por-NPs containing 0.3% porphyrin-lipid of the total moles of lipid (0.3 mol %) were used for *in vitro* studies. For imaging studies, the highest porphyrin-lipid content consisted of the discoidal 30 mol % Por-NPs which contain 30% porphyrin-lipid of the total moles of lipid.

For nanoparticle preparation (Supplemental Table S7), lipid films were made by adding 1,2-dimyristoyl-sn-glycero-3-phosphocholine (DMPC, Sigma Aldrich) into a round bottom flask. For CO porphyrin-containing formulations, cholesterol oleate (Sigma Aldrich) and pyropheophorbide-a-lipid [1, 2] were additionally added to the flask in quantities described in Supplemental Table S7 (CO 20 mol/mol% total lipid, porphyrin 30 mol% of total lipid). Chloroform (2-3 mL) was added to the flask to dissolve the lipids and an additional 2 mL of chloroform was used to wash down the sides of the flask to ensure all solids were dissolved. A lipid film was created by rotating the flask manually under a steady flow of nitrogen until a thick viscous coating was formed around the bottom third of the flask. This film was placed under strong nitrogen flow for a minimum of one hour before transferring to a desiccator equipped with a vacuum. The flask was then vacuum dried overnight. The next day sterile PBS (Sigma Aldrich) was added to the dried lipid film. A suspension was created by exposing the flask to water bath sonication (48^o^C) until the lipid film was suspended completely and homogeneously into the PBS, yielding a suspension that was beginning to clarify (approximately 30 minutes). The suspension was transferred to 15 mL falcon tubes (2 mL suspension per tube) then sonicated with a Bioruptor® probe sonicator for 60 cycles (30 s on/30 s off, low power) at 40^o^C. The lipid suspension was allowed to cool passively to room temperature. Subsequently 0.5 mL of a 13.3 or 26.67 mg/mL R4F peptide solution (see Supplemental Table 7) in sterile PBS was added to each tube in a drop-wise fashion at a 7.34:1 lipid:peptide molar ratio. The addition of R4F makes the solution become translucent. After the dropwise addition, the individual solutions were mixed via pipette prior to combining all 15 mL into a single falcon tube. The falcon tube was sealed with parafilm, protected from light with aluminum foil and rotated for 12-24 h at 4^o^C. The tube was then centrifuged at 4^o^C for 60 min at 4000x*g*. This yields a pellet. The supernatant was syringe filtered in a biosafety cabinet (0.2 micron syringe filter) into a sterile falcon tube, yielding the desired particles, which were subsequently characterized by UPLC-MS, and transmission electron microscopy (TEM). Particle solutions were aliquoted into cryotubes and flash frozen in liquid nitrogen before storage at -80°C, and thawed before use in a 37^o^C water bath.

**Supplemental Table S7: Lipid film compositions for nanoparticle formulations**

| **Particle** | **DMPC** | **CO** | **Pyro-pheophorbide-a-lipid** | **PBS for lipid film hydration** | **Total R4F peptide** | **R4F peptide concentration / mg·mL^-1^** |
| --- | --- | --- | --- | --- | --- | --- |
| **Porphyrin-NP discoidal (30%)** | 30.2 mg  44.5 µmol | - | 19.3 mg  19.1 µmol | 6 mL | 20 mg  8.64 µmol | 13.33 |
| **Porphyrin-NP CO-loaded (30%)** | 30.2 mg  44.5 µmol | 8.27 mg  12.7 µmol | 19.3 mg  19.1 µmol | 6 mL | 20 mg  8.64 µmol | 13.33 |
| **Porphyrin-NP discoidal (0.3%)** | 22 mg  32.4 µmol | - | 0.0987 mg  97.9 nmol | 5 mL | 5 mg  2.16 µmol | 5 |
| **Porphyrin-NP CO-loaded (0.3%)** | 22 mg  32.4 µmol | 4.3 mg  6.5 µmol | 0.0987 mg  97.9 nmol | 5 mL | 5 mg  2.16 µmol | 5 |

**Optical Characterization**

As previously described^3^, optical characterization of solutions containing either intact or disrupted particles was conducted using UV-Vis spectrometry (Varian 50 Bio). All intact particle measurements were conducted in PBS. Discoidal and CO-loaded Por-NPs were disrupted in methanol.

**Reconstituted high-density lipoprotein (rHDL)**

Apolipoprotein A-I (ApoA-I), the main protein component of native HDL, was isolated from pooled donated plasma samples from healthy humans obtained from the Australian Red Cross (Supply Agreement 14-02NSW-04) by ultracentrifugation and anion-exchange chromatography, as described previously [4-7]. Discoidal rHDL was prepared by complexing purified lipid free apoA-I with phospholipid 1-palmitoyl-2-linoleoyl-phosphatidycholine (PLPC) at an initial PLPC: apoA-I ratio of 100:1. The rHDL was filter sterilised prior to use in the cholesterol efflux experiments. The final apoA-I concentration was determined using the BCA assay.

**Transmission Electron Microscopy**

Transmission electron microscopy (TEM) data acquisition and analysis was conducted as per Rajora *et al.* [3]. Briefly, transmission electron microscopy was conducted with a FEI Tecnai T20 electron microscope at the Nanoscale Biomedical Imaging Facility, Peter Gilgan Centre for Research and Learning. Samples were diluted 20-50x in ddH_2_O and loaded onto charged carbon coated grids. The grids were stained with 2% uranyl acetate negative stain and imaged at direct magnification of 60,000 to 100,000x. The mean particle size and polydispersity index (PDI) were calculated based on three batches of each type of nanoparticle, using multiple views of TEM images (50–300 particles per image, with a minimum of three images per batch), analyzed through ImageJ.

**Real time-qPCR**

Total RNA was extracted from iBMDMs and murine aortic arches with TRI-reagent (Sigma-Aldrich). RNA concentration was quantified using nanodrop spectrophotometer (Thermofisher Scientific) with the purity of the samples assessed using the absorbance ratio (A260/A280 ~2.0 for RNA). Then 1400-1845 ng (iBMDMs) or 500ng (aortic arch) of total RNA was reverse transcribed to cDNA using iScript Reverse Transcriptase Supermix (Biorad). Primers for inflammatory and housekeeper genes (Table S2) were used to measure gene expression changes by qPCR. Gene expression was calculated using the ^ΔΔ^*Ct* method referenced to the housekeeper gene *Rplp0* or *Beta-2 microglobulin* (*B2m).*

**Histology**

**Embedding and sectioning of aortic sinus and tandem stenosis carotid segment I**

A transverse cut was made through the entire heart positioned perpendicular to the base of the two atria and ~1 mm below and proximal side embedded in optimal cutting temperature (OCT) compound (TissueTek). For the tandem stenosis model [8], Segment I of the carotid (region below proximal suture) was embedded upright in OCT. To section the aortic sinus, embedded hearts were trimmed until the first leaflet emerged and sections collected from this point. At least sixty 7 µm sections of the OCT-embedded aortic sinus were collected continuously on a cryostat (Leica CM3050S) and mounted onto microscope slides (Superfrost Plus). Additionally, the carotid artery segment of the tandem stenosis model that develops unstable plaque, tandem stenosis Segment I, was serially sectioned at 6 µm [8]. Segment I was sectioned from the proximal suture for a length of approximately 2.3 mm at ~96 µm intervals.

**Hematoxylin and Eosin (H&E) staining**

Fresh frozen OCT-embedded sections were fixed with 10% neutral buffered formalin then stained using a standard H&E staining protocol. Briefly, sections were stained in Mayer's Hematoxylin, 0.3% acid ethanol, Scott’s tap water and Eosin. Sections were dehydrated and mounted in Dibutylphthalate Polystyrene Xylene (DPX, Sigma-Aldrich Inc., MO, USA).

**Masson’s Trichrome staining**

Fresh frozen OCT-embedded sections were fixed in 4% PFA before incubation in Bouin’s fluid overnight at room temperature. Masson’s trichrome staining was conducted using a Trichrome Stain Kit following the manufacturer’s instructions (ab150686, Abcam). Sections were dehydrated and mounted in DPX.

**Oil red O staining**

Fresh frozen OCT-embedded sections were fixed in 10% neutral buffered formalin. Sections were washed with 60% v/v isopropanol then stained in Oil red O solution (0.6% w/v oil red O, 60% v/v isopropanol) and differentiated in 60% v/v isopropanol. Sections were mounted with Aquatex (Merck-Millipore).

**Alpha-smooth muscle actin (α-SMA)** **immunohistochemistry**

OCT-embedded sections were fixed in 2% PFA for 10 min. Sections were blocked with 10% goat serum (Sigma-Aldrich Inc., MO, USA), incubated with α-SMA conjugated to alkaline phosphatase antibody (clone 1A4, A5691, Sigma-Aldrich Inc., MO, USA) diluted 1:100, then alkaline phosphatase development with Vector Red substrate kit (Vector Laboratories, Burlingame, CA, USA) following the manufacturer’s instructions. Sections were dehydrated and mounted in DPX.

**CD68 immunofluorescence staining**

Sections were fixed with 2% PFA for 10 min and then washed with 0.1 M glycine solution. Sections were blocked with 5% goat serum and incubated with anti-CD68 (rat monoclonal, Clone FA-11, IgG2a, MCA1957GA, Biorad) diluted 1:250 or Rat IgG2a κ Isotype Control (BD Biosciences) diluted 1:1250 then Donkey anti-Rat IgG (H+L) Alexa Fluor 488 (AF-488) secondary antibody (A-21208, Invitrogen) diluted 1:2000. Sections were mounted with VECTASHIELD® Antifade Mounting Medium with DAPI (Vector Laboratories).

**TER-119 immunohistochemistry**

Fresh frozen OCT-embedded sections were fixed in ice-cold acetone. Sections were incubated in 3% v/v H_2_O_2,_ blocked with 10% normal horse serum then avidin and biotin. Sections were incubated with TER-119 biotinylated rat anti-mouse monoclonal antibody (Thermofisher, MA, USA) diluted 1:400. TER-119 was detected with Vectastain Elite ABC HRP kit according to manufacturer’s instructions (Vector Laboratories, CA, USA). Sections were dehydrated and mounted in DPX.

**Microscopy**

Stained sections were imaged under the Axiolab microscope attached to a camera (Zeiss) or imaged with Nanozoomer digital slide scanner (Hamamatsu). Fluorescence in aortic sinus sections was detected using the 20x objective on an Axio Scan.Z1 slide scanner microscope (Zeiss) with CD68-AF488 staining and porphyrin-lipid detected on the AF488 and Cy5 fluorescence channels respectively. Fluorescence in segment I in the carotid artery of the tandem stenosis model was detected using the 10x objective on the Eclipse NiE fluorescence microscope (Nikon) with CD68-AF488 staining and porphyrin-lipid detected on the FITC and Cy5 fluorescence channels respectively.

**Histological image analysis**

Image analysis of tissue sections was performed using Image Pro-Premier 9.2 (Media Cybernetics) software. For the aortic sinus, three H&E sections spanning the aortic sinus that contained three leaflets were analyzed per animal and expressed as average total lesion area. The area of histological staining from a mid-point section was calculated as a percentage of the selected total lesion area (% of plaque area) for histological, immunochemical and immunofluorescence analyses.

For tandem stenosis Segment I, plaque/lesion area was measured in H&E-stained sections in three separate regions spanning the length of the vessel at 0-384 μm, 768-1152 μm and 1920-2304 μm from the proximal suture. Four sections 96 µm apart in the mid-point region of Segment I (approx. 768-1152 μm from proximal suture) were assessed for histological and immunochemical analyses.

**Plasma lipid analysis**

Triglycerides, total cholesterol, LDL-cholesterol and HDL-cholesterol were measured enzymatically in plasma using commercially available kits (Wako diagnostics). For triglycerides, 2 µL of plasma was using for the assessment, according to manufacturer’s instructions, with color change to blue measured at 600 nm absorbance. The same protocol was followed for total cholesterol, but the plasma was first diluted 1:1 with distilled water. To determine the HDL-cholesterol concentration, 20 µL of plasma was mixed with 20 µL of precipitation reagent from HDL-C kit (Wako diagnostics), then incubated for 10 min at room temperature before centrifugation at 3000 rpm for 15 min. 10 µL of the supernatant was assayed with the total cholesterol kit to determine HDL-C concentration. LDL-C concentration was calculated by subtracting HDL-C concentrations from total cholesterol concentration.

# References

1. Lovell, J.F., C.S. Jin, E. Huynh, H. Jin, C. Kim, J.L. Rubinstein, W.C.W. Chan, W. Cao, L.V. Wang, and G. Zheng, Porphysome nanovesicles generated by porphyrin bilayers for use as multimodal biophotonic contrast agents. Nature Materials, 2011. **10**: p. 324.

2. Cui, L., Q. Lin, C.S. Jin, W. Jiang, H. Huang, L. Ding, N. Muhanna, J.C. Irish, F. Wang, J. Chen, and G. Zheng, A PEGylation-Free Biomimetic Porphyrin Nanoplatform for Personalized Cancer Theranostics. ACS Nano, 2015. **9**(4): p. 4484-95.

3. Rajora, M.A., L. Ding, M. Valic, W. Jiang, M. Overchuk, J. Chen, and G. Zheng, Tailored theranostic apolipoprotein E3 porphyrin-lipid nanoparticles target glioblastoma Chemical Science, 2017. **8**(8): p. 5371-5384.

4. Tan, J.T.M., H.C.G. Prosser, L.L. Dunn, L.Z. Vanags, A. Ridiandries, T. Tsatralis, L. Leece, Z.E. Clayton, S.C.G. Yuen, S. Robertson, Y.T. Lam, D.S. Celermajer, M.K.C. Ng, and C.A. Bursill, High-Density Lipoproteins Rescue Diabetes-Impaired Angiogenesis via Scavenger Receptor Class B Type I. Diabetes, 2016. **65**(10): p. 3091-3103.

5. Bursill, C.A., M.L. Castro, D.T. Beattie, S. Nakhla, E. van der Vorst, A.K. Heather, P.J. Barter, and K.A. Rye, High-density lipoproteins suppress chemokines and chemokine receptors in vitro and in vivo. Arterioscler Thromb Vasc Biol, 2010. **30**(9): p. 1773-8.

6. van der Vorst, E.P., L.Z. Vanags, L.L. Dunn, H.C. Prosser, K.A. Rye, and C.A. Bursill, High-density lipoproteins suppress chemokine expression and proliferation in human vascular smooth muscle cells. FASEB J, 2013. **27**(4): p. 1413-25.

7. Tsatralis, T., A. Ridiandries, S. Robertson, L.Z. Vanags, Y.T. Lam, J.T.M. Tan, M.K.C. Ng, and C.A. Bursill, Reconstituted high-density lipoproteins promote wound repair and blood flow recovery in response to ischemia in aged mice. Lipids in Health and Disease, 2016. **15**(1): p. 150.

8. Chen, Y.C., A.V. Bui, J. Diesch, R. Manasseh, C. Hausding, J. Rivera, I. Haviv, A. Agrotis, N.M. Htun, J. Jowett, C.E. Hagemeyer, R.D. Hannan, A. Bobik, and K. Peter, A novel mouse model of atherosclerotic plaque instability for drug testing and mechanistic/therapeutic discoveries using gene and microRNA expression profiling. Circ Res, 2013. **113**(3): p. 252-65.
